# SIX2‐Mediated Microglial M2 Polarization and Exosomal miR‐3470b Delivery Protect Dopaminergic Neurons in Parkinson's Disease

**DOI:** 10.1002/cns.70756

**Published:** 2026-02-17

**Authors:** Jia‐shuo Kan, Xia‐yin Cao, Yu‐xin Ye, Xin‐xing Huang, Guo‐jing Sun, Jin Gao

**Affiliations:** ^1^ Department of Neurobiology and Cellular Biology Xuzhou Medical University Xuzhou Jiangsu China

**Keywords:** exosomes, miR‐3470b, neuroinflammation, Parkinson's disease, SIX2

## Abstract

**Aims:**

Neuroinflammation driven by dysregulated microglial activation exacerbates dopaminergic neuron loss in Parkinson's disease (PD). This study investigated whether the transcription factor SIX2 mitigates neuroinflammation and provides neuroprotection by promoting microglial M2 polarization and exosome‐mediated communication.

**Methods:**

Using LPS‐stimulated BV2 microglia and MPTP‐induced mouse models of PD, we systematically investigated the role of SIX2. Gain‐ and loss‐of‐function approaches for SIX2, DDIT4, miR‐3470b, and GREM1 were combined with ChIP, RNA‐seq, exosome isolation/transfer, and behavioral tests to analyze the SIX2‐DDIT4‐autophagy axis and the exosomal miR‐3470b/GREM1/TGF‐β pathway.

**Results:**

RNA‐seq and ChIP‐qPCR revealed that SIX2 transcriptionally activated DDIT4. This led to mTOR inhibition and autophagy induction, driving a shift in microglial phenotype from pro‐inflammatory M1 to protective M2. Consequently, M2‐polarized microglia released exosomes highly enriched in miR‐3470b, as identified by miRNA sequencing. Upon internalization by dopaminergic neurons, miR‐3470b directly bound to and suppressed GREM1, which in turn potentiated TGF‐β signaling activity. Ultimately, this SIX2‐initiated cascade rescued neuronal apoptosis and restored motor coordination in both cellular and animal models of PD.

**Conclusion:**

SIX2 promotes microglial M2 polarization via the DDIT4/mTOR/autophagy axis and mediates neuroprotection through exosomal miR‐3470b targeting of GREM1/TGF‐β signaling, revealing novel therapeutic targets for PD immunotherapy.

## Graphical abstract

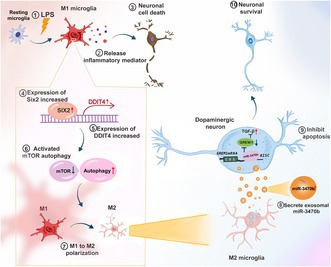

## 1 Introduction

Parkinson's Disease (PD) is a neurodegenerative disorder characterized by the progressive degeneration and death of dopaminergic neurons in the substantia nigra pars compacta (SNpc) of the midbrain. The clinical manifestations of PD include motor symptoms, such as resting tremor, muscle rigidity, bradykinesia, and postural instability. Current pharmacological treatments for PD primarily aim to increase the level of brain dopamine, for example, the use of levodopa and dopamine receptor agonists. The drug therapies are unable to fundamentally slow or halt the progressive death of dopaminergic neurons [1, 2, 3]. Thus, identifying novel therapeutic targets to decelerate or prevent the degeneration of dopaminergic neurons remains a major challenge in PD therapy.

Neuroinflammation plays a crucial role in the pathogenesis and progression of PD [4, 5, 6]. As the resident macrophages in the brain, microglia are closely associated with the onset and development of PD [7, 8, 9]. Activated microglia exhibit a dual phenotype: (i) the “classical activation” M1 phenotype that is pro‐inflammatory and neurotoxic; and (ii) the “alternative activation” M2 phenotype that is anti‐inflammatory and neuroprotective [10]. The M1 microglia release pro‐inflammatory factors, such as TNF‐α, IL‐6, and iNOS, leading to neuronal injury and the release of damage‐associated molecules, which further amplify the inflammatory response and accelerate PD progression. In contrast, the M2 microglia produce high levels of ARG‐1, IL‐10, and CD206, and growth factors, for example, vascular endothelial growth factor and brain‐derived neurotrophic factor, which promote the repair of surrounding cells and the homeostatic environment in the brain [11]. Under certain conditions, M1/M2 microglia can interconvert [12]. Therefore, the shift of microglia from the M1 to the M2 is crucial in alleviating or reversing the progressive death of dopaminergic neurons in PD.

Given the critical role of neuroinflammation in PD progression, identifying key regulators of microglial activation and polarization is essential. One such potential regulator is the transcription factor Sine oculis homeobox homolog 2 (SIX2), a developmental transcription factor largely silenced in healthy adults [13, 14]. However, upon tissue injury or stress, SIX2 is rapidly re‐activated and exerts potent anti‐inflammatory effects, as demonstrated in lipopolysaccharide (LPS)‐induced differentiated macrophages [15]. In the central nervous system, our previous study showed that SIX2 expression is significantly upregulated in inflammatory microglia, where it inhibits LPS‐induced neuroinflammation and protects co‐cultured dopaminergic neurons [16]. Despite these findings, the precise mechanisms by which SIX2 exerts these beneficial effects remain unknown.

In addition to its direct anti‐inflammatory effects, SIX2 may modulate intercellular communication through exosomes, nanoscale extracellular vesicles (30–150 nm) that carry non‐coding RNAs, proteins, and lipids. In the brain, microglia‐derived exosomes play crucial roles in regulating neuroinflammation and neuronal survival [17, 18, 19]. Notably, exosomes secreted by anti‐inflammatory (M2) microglia have been shown to carry neuroprotective cargo, such as specific miRNAs, and can promote neuronal survival upon uptake [20, 21]. Notably, exosomes protect their cargo, particularly microRNAs (miRNAs), from degradation, enabling efficient intercellular communication [22, 23, 24]. Emerging evidence suggests that regulating exosomal miRNA profiles potentially mediates intercellular crosstalk in neurodegenerative diseases [25, 26]. This raises an intriguing question: do microglia overexpressing SIX2 secrete exosomes carrying specific miRNAs, and can these exosomes mediate neuroprotection in co‐cultured dopaminergic neurons?

Here, we aim to elucidate the molecular and cellular mechanisms by which SIX2 modulates LPS‐induced inflammatory injury in microglia, particularly focusing on its role in directing the polarization of microglia from the M1 to the M2 phenotype. Furthermore, we seek to determine whether SIX2‐mediated microglial polarization leads to the secretion of exosomes carrying specific miRNAs, and whether these exosomes can confer neuroprotection to co‐cultured dopaminergic neurons. Addressing this question could provide novel insights into the role of SIX2 in modulating neuroinflammation and offer new therapeutic strategies for PD.

## 2 Results

### 2.1 SIX2 Modulates BV2 Microglial Cells Polarization and Protects Dopaminergic Cells in LPS‐Induced Cytotoxicity

**FIGURE 1:**
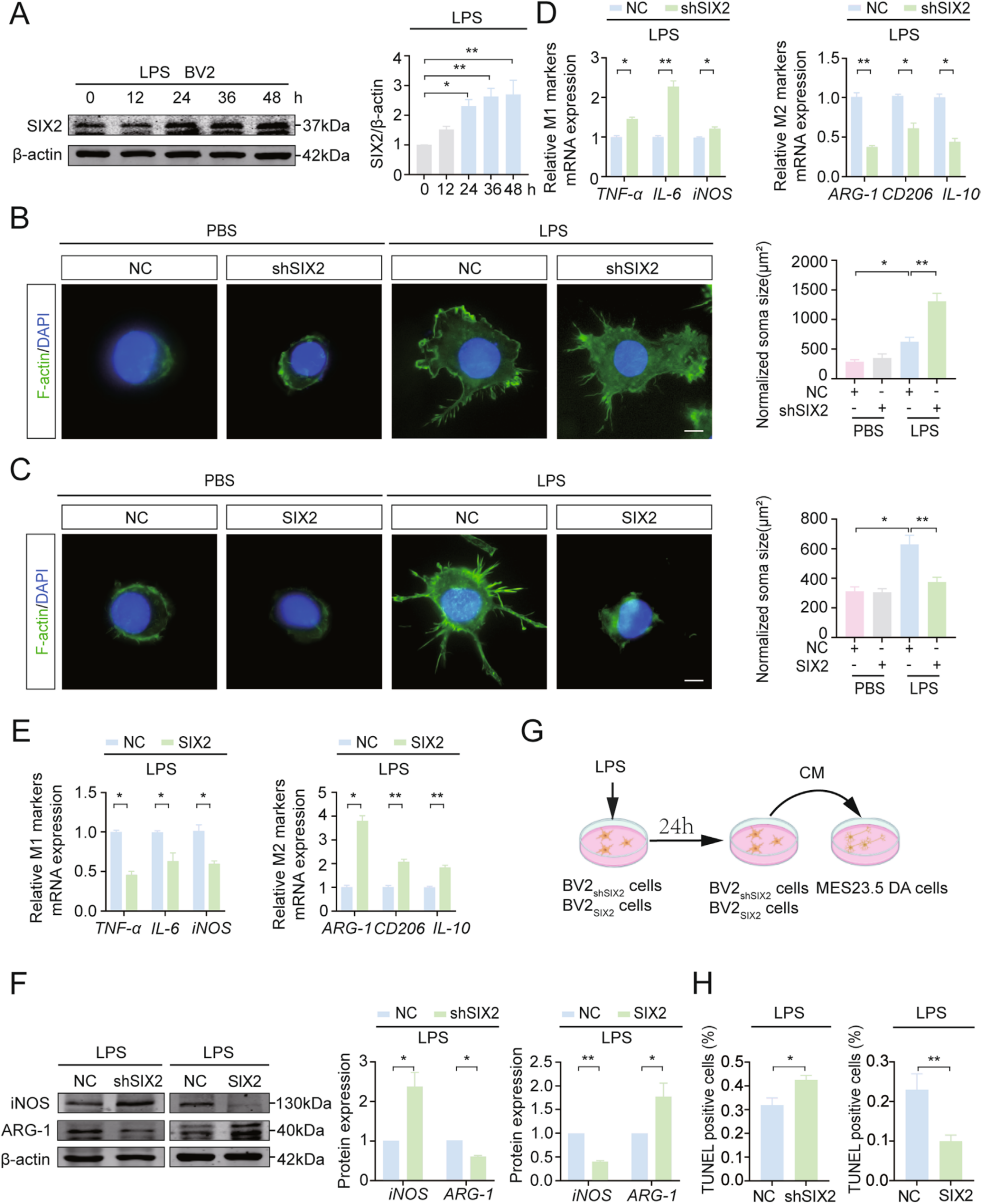
SIX2 Promotes M1 to M2 polarization of LPS‐induced BV2 microglial cells to protect MES23.5 DA cells. (A) WB analysis of SIX2 protein expression in BV2 cells treated with 100 ng/mL LPS for various durations. (B, C) Immunofluorescence staining (F‐Actin, green) showing the effect of SIX2 knockdown and overexpression on BV2 cell somatic size. Scale bar = 10 μm. (D, E) After 24 h of LPS(100 ng/mL) treatment, RT‐qPCR analysis of M1 and M2 microglial markers in BV2 cells with knockdown (BV2_shSIX2_) and overexpression (BV2_SIX2_) of SIX2 after 24 h LPS treatment. (F) WB analysis of iNOS and ARG‐1 in BV2_shSIX2_ and BV2_SIX2_ microglial cells after 24 h LPS treatment. (G, H) Schematic of the co‐culture system and its effect on neuronal apoptosis. Conditioned medium (CM) from LPS‐treated BV2shSIX2 or BV2SIX2 was applied to MES23.5 DA cells for 24 h. Neuronal apoptosis was assessed by TUNEL staining (*p < 0.05, **p < 0.01, *n* = 3).

SIX2 expression in BV2 microglial cells (BV2 cells) was significantly increased by over 2‐fold after LPS treatment in a time‐dependent manner (Figure 1A). Immunofluorescence analysis of primary microglia from newborn SD rats confirmed that over 98% of cells were IBA1‐positive (IBA1+) (Figure S1A), and SIX2 fluorescence intensity increased significantly after LPS treatment (Figure S1B). To determine if SIX2 promotes microglial polarization from M1 to M2, BV2 cells were transfected with shSIX2 (knockdown) or SIX2‐coding sequence (overexpression) and treated with LPS for 24 h. SIX2 expression was significantly reduced in the knockdown group (Figure S2A) and increased in the overexpressed group (Figure S2B). Immunofluorescence staining revealed that LPS‐induced BV2 cell body enlargement, which was further exacerbated by SIX2 knockdown (Figure 1B) but restored to control levels by SIX2 overexpression (Figure 1C). In the knockdown group, mRNA levels of M1 markers (*TNF‐α*, *IL‐6*, *iNOS*) were significantly increased, while M2 markers (*ARG‐1, CD206, IL‐10*) were decreased (Figure 1D). Conversely, SIX2 overexpression reversed these effects (Figure 1E). Protein analysis confirmed that SIX2 knockdown increased iNOS and decreased ARG‐1 expression, while overexpression had the opposite effect (Figure 1F). Consistently, ELISA analysis of the culture medium showed that SIX2 knockdown reduced the secretion of IL‐10 and increased that of IL‐6, whereas overexpression had the opposite effects (Figure S3A,B). These results indicate that SIX2 promotes microglial polarization from M1 to M2.

Since LPS‐activated microglia damage surrounding neurons by releasing inflammatory factors, we next investigated whether SIX2‐regulated microglia could protect neurons from this damage. The dopaminergic cell line MES23.5 (MES23.5 cells) was cultured in conditioned medium from LPS‐treated BV2 cells for 24 h with SIX2 knockdown (BV2_shSIX2_ cells) or overexpression (BV2_SIX2_ cells) (Figure 1G). Apoptosis rates of MES23.5 cells were significantly higher in the SIX2 knockdown group but lower in the overexpression group (Figure 1H). These findings demonstrate that SIX2 overexpression in BV2 cells suppresses LPS‐induced cytotoxicity and protects co‐cultured MES23.5 cells.

### 2.2 Overexpression of SIX2 in Microglia Promotes M1‐To‐M2 Polarization in an LPS‐Induced Neuroinflammatory Mice

**FIGURE 2:**
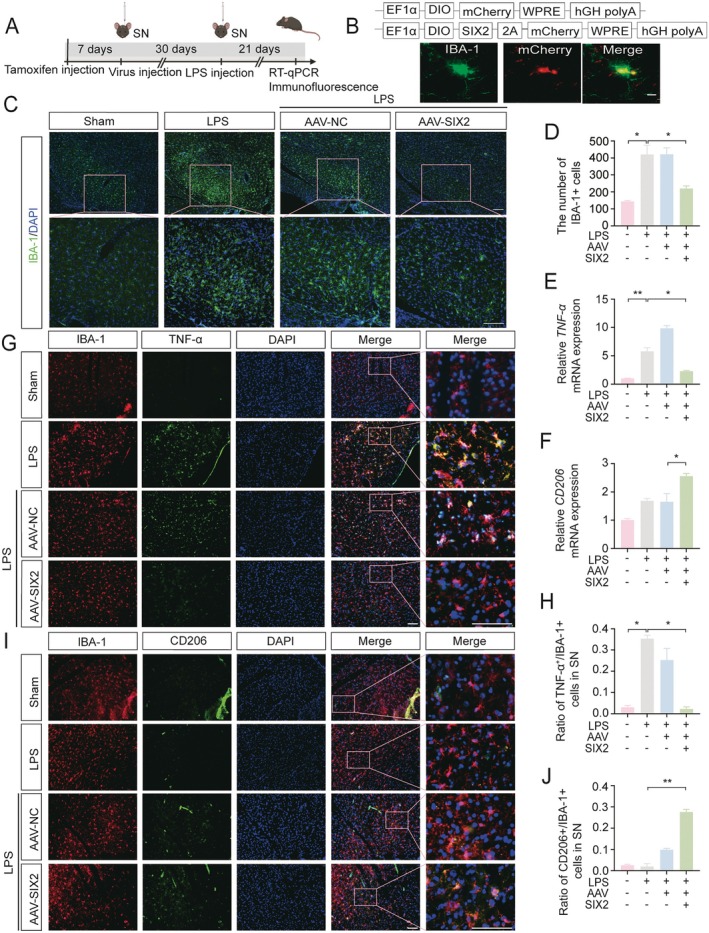
Overexpression of SIX2 in microglia promotes M2 polarization in LPS‐induced mice. (A) Experimental procedure: Cx3cr1‐CreERT2 mice were treated with tamoxifen, followed by AAV‐SIX2 injection into the right SNpc 7 days later. LPS(0.25 mg/kg in 1 μL PBS) [16] was injected into the right SNpc of the mice 30 days later, and mice were sacrificed 21 days post‐LPS injection for RT‐qPCR and immunofluorescence staining. (B) Top: Flowchart for overexpressing SIX2 with AAV‐SIX2 in microglia. Bottom: Co‐labeling images demonstrating successful overexpression of SIX2 (visualized by mCherry expression, red) in IBA1+ microglia. Scale bar =10 μm. (C, D) Immunofluorescence staining with IBA1 (green) and statistical analysis to quantify IBA1+ microglia in the SNpc of mice, Scale bar = 200 μm. (E, F) RT‐qPCR results for the *TNF‐α* and *CD206* levels in the midbrain of mice. (G, H) Immunofluorescence staining with IBA1 (red) and TNF‐α (green) antibodies, and statistical analysis of TNF‐α + microglia relative to IBA1+ microglia in the SNpc. Scale bar = 100 μm. (I, J) Immunofluorescence staining with IBA1 (red) and CD206 (green) antibodies, and statistical analysis of CD206+ microglia relative to IBA1+ microglia in the SNpc. Scale bar = 100 μm (*p < 0.05, **p < 0.01, *n* ≥ 6).

SIX2 was specifically overexpressed in nigral microglia using AAV‐SIX2‐mcherry (Figure 2A). Immunofluorescence confirmed co‐localization of the microglial‐specific marker IBA1 with mcherry‐labeled SIX2 (Figure 2B). LPS treatment increased the number of IBA1+ cells, which was restored to control levels by SIX2 overexpression (Figure 2C,D). LPS challenge also elevated *TNF‐α* mRNA levels in the midbrain, an effect that was abolished by SIX2 overexpression (Figure 2E). Similarly, SIX2 overexpression reversed the LPS‐mediated decrease in *CD206* mRNA levels (Figure 2F). It is noteworthy that LPS alone induced a moderate increase in CD206 expression compared to the control, which may reflect the initiation of an endogenous compensatory or repair response to inflammation. Strikingly, SIX2 overexpression robustly amplified this response, elevating CD206 to levels significantly above those in the LPS‐only group. Immunofluorescence staining of TNF‐α or CD206 with IBA1 was used to quantify M1 and M2 microglia among activated microglia. The proportion of TNF‐α + microglia was significantly higher in the LPS‐treated group compared to controls (Figure 2G,H), whereas CD206+ microglia were significantly reduced. As expected, SIX2 overexpression reversed these effects (Figure 2I,J). These results demonstrate that SIX2 overexpression in microglia promotes M1‐to‐M2 polarization in the substantia nigra and inhibits LPS‐induced neuroinflammation.

### 2.3 SIX2 Promotes M2 Polarization of LPS‐Induced Microglial Cells by Upregulating DDIT4

**FIGURE 3:**
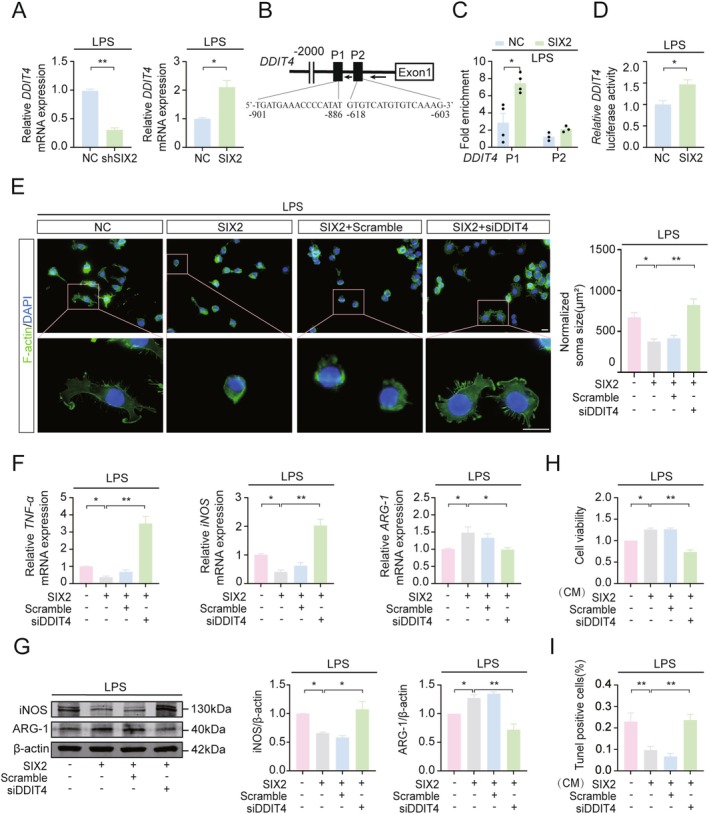
SIX2 Promotes M2 polarization of LPS‐induced BV2 cells by upregulating DDIT4. (A) RT‐qPCR analysis of *DDIT4* mRNA levels in LPS‐treated BV2_shSIX2_ or BV2_SIX2_ cells. (B) JASPAR prediction of SIX2 binding sites on the *DDIT4* promoter. (C) ChIP‐qPCR analysis of SIX2 binding to the *DDIT* promoter region in LPS‐treated BV2_SIX2_ cells. (D) Dual‐luciferase reporter assay of SIX2 binding to *DDIT4* promoter in LPS‐treated BV2_SIX2_ cells. (E) Immunofluorescence staining of F‐Actin (green) to assess somatic size in LPS‐treated BV2_SIX2_ cells with DDIT4 silenced, Scale bar = 10 μm. (F) RT‐qPCR analysis of *TNF‐α*, *iNOS* and *ARG1* in LPS‐treated BV2_SIX2_ cells with DDIT4 silenced. (G) WB analysis of iNOS and ARG1 levels in LPS‐treated BV2_SIX2_ cells with DDIT4 silenced. (H, I) Conditioned medium from LPS‐treated BV2_SIX2_ cells with DDIT4 silenced was co‐cultured with MES23.5 DA cells. Cell viability and apoptosis were assessed by CCK‐8 and TUNEL assays, respectively (*p < 0.05, **p < 0.01, *n* = 3).

RNA‐seq analysis of LPS‐treated BV2_shSIX2_ cells revealed that SIX2 knockdown upregulated 72 genes and downregulated 116 genes (Figure S4A). Using ChIP‐atlas to screen for SIX2 target genes and intersecting the results with RNA‐seq data, DDIT4 was identified as a key downstream target (Figure S4B). RT‐qPCR further confirmed that DDIT4 expression was markedly reduced in LPS‐treated BV2_shSIX2_ cells but upregulated in LPS‐treated BV2_SIX2_ cells (Figure 3A). SIX2 overexpression also increased DDIT4 expression in BV2 cells (Figure S5A). ChIP‐qPCR confirmed that SIX2 bound to the DDIT4 promoter at −901 to −896 bp (P1) and −618 to −603 bp (P2), with stronger affinity for P1 (Figure 3B,C). Dual‐luciferase reporter assays further demonstrated that SIX2 significantly enhanced DDIT4 transcriptional activity in LPS‐treated BV2_SIX2_ cells (Figure 3D). These results indicate that SIX2 directly binds to the *DDIT4* promoter and upregulates its expression in LPS‐treated BV2 cells.

To determine the role of DDIT4 in SIX2‐mediated effects, DDIT4 was silenced using siRNA (Figure S5B,C). DDIT4 silencing abolished the ability of SIX2 to inhibit LPS‐induced BV2 cell enlargement (Figure 3E). Additionally, SIX2 overexpression significantly decreased *TNF‐α* and *iNOS* mRNA levels while increasing *ARG‐1* mRNA levels in LPS‐treated BV2 cells, effects reversed by DDIT4 silencing (Figure 3F). Similarly, DDIT4 silencing abolished SIX2‐mediated changes in iNOS and ARG‐1 protein expression (Figure 3G). CCK8 assays revealed that SIX2 promoted the viability of LPS‐activated BV2 cells, an effect abolished by DDIT4 silencing (Figure 3H). TUNEL assays further confirmed these findings (Figure 3I).

Similar results were observed in primary microglia. Lentivirus‐mediated SIX2 overexpression increased SIX2 levels (Figure S5D), while immunofluorescence confirmed reduced DDIT4 expression in SIX2‐overexpressed, LPS‐treated microglia (Figure S5E). DDIT4 knockdown reversed SIX2‐mediated effects on microglial cell size (Figure S5F) and the expression of iNOS (Figure S5G) and ARG‐1 (Figure S5H). Together, these findings demonstrate that SIX2 promotes M1‐to‐M2 polarization in LPS‐induced microglia by upregulating DDIT4 expression.

### 2.4 SIX2/DDIT4 Signaling Promotes M2 Polarization of BV2 Cells by Inhibiting Autophagy

**FIGURE 4:**
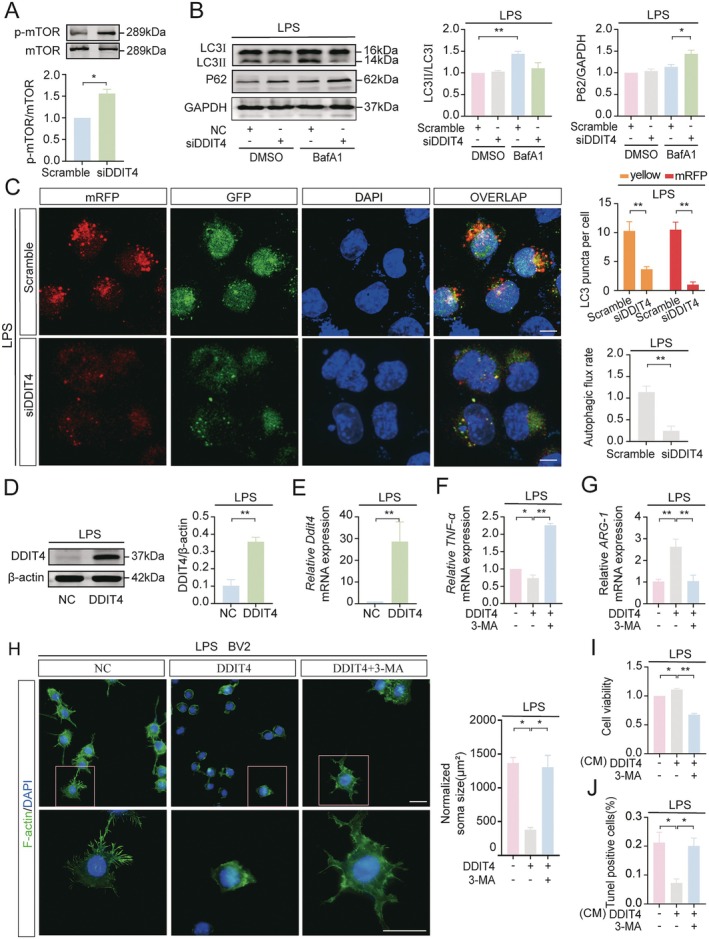
DDIT4 Promotes M2 Polarization of LPS‐treated BV2 cells by inhibiting mTOR and activating autophagy. (A, B) WB analysis of mTOR phosphorylation, LC3II/LC3I, and P62 levels in LPS‐treated BV2_SIX2_ cells with DDIT4 silenced. (C) Autophagic flux detected by fluorescence microscopy in LPS‐treated BV2_SIX2_ microglial cells with DDIT4 silenced. Scale bar = 10 μm. (D, E) WB and RT‐qPCR analyses of DDIT4 overexpression in BV2 microglial cells. (F, G) BV2 microglial cells overexpressing DDIT4 were pre‐treated with 3‐MA(5 mM) for 1 h followed by LPS treatment for 24 h. RT‐qPCR analysis of *TNF‐α* (F) and *ARG‐1* (G) mRNA levels. (H) Immunofluorescence staining of F‐Actin (green) in BV2 microglial cells, with somatic size quantified using ImageJ. Scale bar = 20 μm (overview) and 10 μm (inset). (I, J) Conditioned medium from BV2 microglial cells was co‐cultured with MES23.5 DA cells. Cell viability (I) and apoptosis (J) were assessed by CCK‐8 and TUNEL assays, respectively. Scale bar = 50 μm (*p < 0.05, **p < 0.01, *n* = 3).

DDIT4 is an inhibitor of mTOR [27], a key factor in initiating autophagy [28]. Since autophagy promotes the polarization of BV2 cells from M1 to M2 [29], we hypothesized that DDIT4 promotes M1‐to‐M2 polarization in LPS‐treated BV2 cells by inhibiting mTOR phosphorylation and activating autophagy. To test this, we measured mTOR phosphorylation and the protein levels of LC3II/I and P62 in LPS‐treated BV2_SIX2_ cells with or without DDIT4 silencing. The late‐stage autophagy inhibitor BafA1 was used to block autophagosome‐lysosome fusion. DDIT4 silencing significantly increased mTOR phosphorylation and P62 protein levels while decreasing LC3II/I levels (Figure 4A,B). To assess the impact of DDIT4 on autophagic flux, BV2_SIX2_ cells were transfected with mRFP‐GFP‐LC3 lentivirus, and autophagic flux was measured after LPS treatment with or without DDIT4 silencing. Compared to controls, DDIT4 silencing reduced the ratio of red to yellow puncta (LC3 indicator), indicating inhibited autophagic flux (Figure 4C). These results suggest that SIX2/DDIT4 signaling activates autophagy in LPS‐treated BV2 cells by inhibiting mTOR phosphorylation.

Next, DDIT4 was overexpressed in LPS‐treated BV2 cells, and autophagy was inhibited using 3‐MA. Western blot and RT‐qPCR confirmed DDIT4 overexpression (Figure 4D,E). DDIT4 overexpression suppressed LPS‐induced *TNF‐α* mRNA levels and increased *ARG‐1* mRNA levels, effects reversed by 3‐MA (Figure 4F,G). F‐actin immunofluorescence staining revealed that DDIT4 overexpression restored LPS‐induced BV2 cell enlargement, an effect abolished by 3‐MA (Figure 4H). Similarly, CCK8 and TUNEL assays showed that DDIT4 overexpression improved cell viability and reduced LPS‐induced apoptosis, effects reversed by 3‐MA (Figure 4I,J). Together, these results demonstrate that DDIT4 promotes M1‐to‐M2 polarization in LPS‐treated BV2 cells by activating autophagy via inhibition of mTOR phosphorylation.

### 2.5 BV2_SIX2_ Cells Protect MES23.5 Cells via Exosome Secretion

**FIGURE 5:**
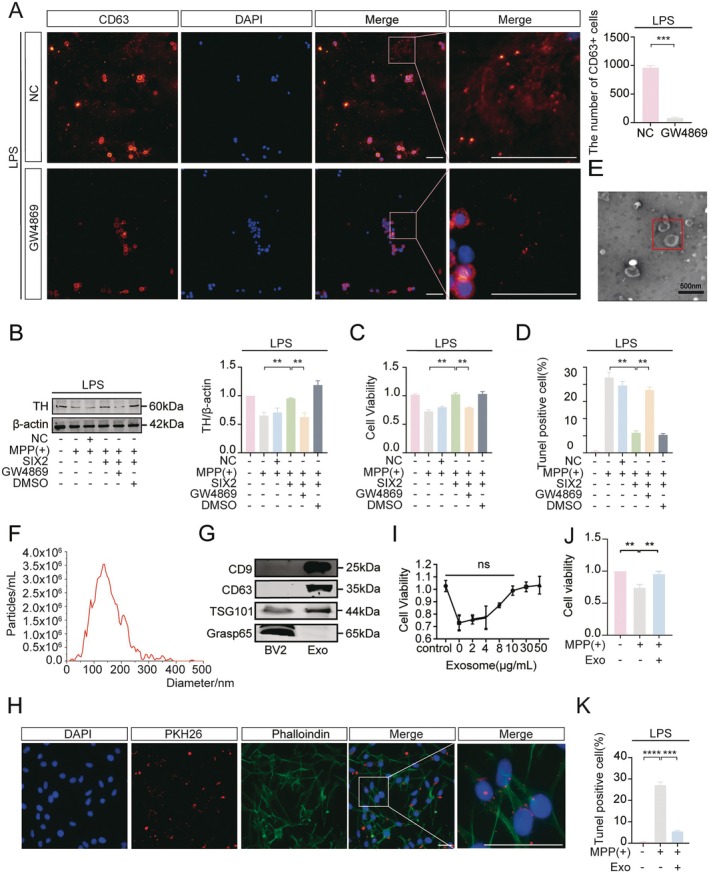
BV2_SIX2_ microglial cells protect MES23.5 DA cells via exosome secretion. (A) Immunofluorescence staining of exosome marker CD63 in BV2_SIX2_ cells treated with GW4869 (exosome inhibitor) (10 μM) and LPS, Scale bar = 50 μm. (B–D) BV2_SIX2_ cells treated with GW4869 and LPS were co‐cultured with MPP(+)‐treated MES23.5 cells. (B) WB analysis of TH expression. (C) CCK‐8 assay for cell viability. (D) TUNEL assay for apoptosis, Scale bar = 100 μm. (E–G) Exosomes from LPS‐treated BV2_SIX2_ cells were analyzed by (E) TEM (scale bar = 500 nm), (F) NTA for size distribution, and (G) WB for exosomal markers (CD9, CD63, TSG101) and Golgi marker Grasp65. (H) PKH‐26‐labeled exosomes (red) from LPS‐treated BV2_SIX2_ cells were internalized by phalloidin‐stained (green) MES23.5 cells, Scale bar = 50 μm. (I–K) MES23.5 cells were treated with exosomes from LPS‐treated BV2SIX2 cells. (I) Different exosome concentrations were tested. (J) CCK‐8 assay showed exosome‐mediated protection. (K) TUNEL staining confirmed anti‐apoptotic effects at 10 μg/mL. Scale bar = 100 μm. (*p < 0.05, **p < 0.01, ***p < 0.001, *n* = 3).

In the central nervous system, microglia secrete exosomes into the extracellular environment, where they regulate brain structure and function by acting on neurons and other glial cells. We next asked whether BV2 cells, polarized from M1 to M2 by SIX2 overexpression, could protect MES23.5 cells via exosome secretion. Immunofluorescence staining showed that GW4869, an exosome inhibitor, reduced CD63+ vesicles in LPS‐treated BV2_SIX2_ cells, indicating decreased exosome secretion (Figure 5A). To test the protective effects of exosomes, the medium from LPS‐treated BV2_SIX2_ cells was co‐cultured with MPP(+)‐treated MES23.5 cells. MPP(+) treatment significantly reduced TH expression in MES23.5 cells, an effect reversed by SIX2 overexpression but blocked by GW4869 (Figure 5B). CCK8 and TUNEL assays further confirmed that SIX2 overexpression improved cell viability and inhibited MPP(+)‐induced apoptosis, effects abolished by GW4869 (Figure 5C,D).

To isolate exosomes, culture supernatants from LPS‐treated BV2_SIX2_ cells were purified by ultracentrifugation. TEM revealed the typical cup‐shaped morphology of exosomes (Figure 5E), and NTA indicated vesicle diameters of 30–120 nm (Figure 5F). WB confirmed high expression of exosomal markers (CD9, TSG101, CD63) in the vesicles, while the Golgi marker GRASP65 was absent (Figure 5G). These results suggest that SIX2 overexpression in LPS‐treated BV2 cells protects MES23.5 cells through exosome secretion.

To assess exosome uptake, vesicles from LPS‐treated BV2_SIX2_ cell supernatants were co‐cultured with MES23.5 cells for 24 h. PKH‐26‐labeled exosomes were observed in the cytoplasm of phalloidin‐stained MES23.5 cells, demonstrating their internalization (Figure 5H). To determine the optimal exosome concentration for neuroprotection, MES23.5 cells were treated with different concentrations of exosomes. At 10 μg/mL, exosomes significantly increased cell viability, and this concentration was used for subsequent experiments (Figure 5I). CCK8 and TUNEL assays confirmed that exosomes from LPS‐treated BV2_SIX2_ cells improved cell viability (Figure 5J) and reduced apoptosis (Figure 5K) in MPP(+)‐treated MES23.5 cells. These results demonstrate that exosomes secreted by LPS‐treated BV2_SIX2_ cells are taken up by MES23.5 cells and exert protective effects.

### 2.6 Exosomal miR‐3470b From LPS‐Treated BV2_SIX2_ Cells Protects MES23.5 Cells From MPP(+) Damage

Exosomes, with their unique membrane structure, protect internal RNA from degradation and serve as key vehicles for intercellular miRNA transfer. To determine if exosomes from LPS‐treated BV2_SIX2_ cells protect MES23.5 cells via miRNA transfer, DROSHA, a key miRNA synthesis enzyme, was silenced in BV2 cells using siRNA. The most efficient siRNA was selected for further study (Figure S6A). Exosomes isolated from these BV2 cells were applied to MPP(+)‐treated MES23.5 cells. Compared to exosomes from control BV2 cells, those from LPS‐treated BV2_SIX2_ cells significantly increased TH protein expression in MES23.5 cells, an effect reversed by DROSHA silencing (Figure S6B).

**FIGURE 6:**
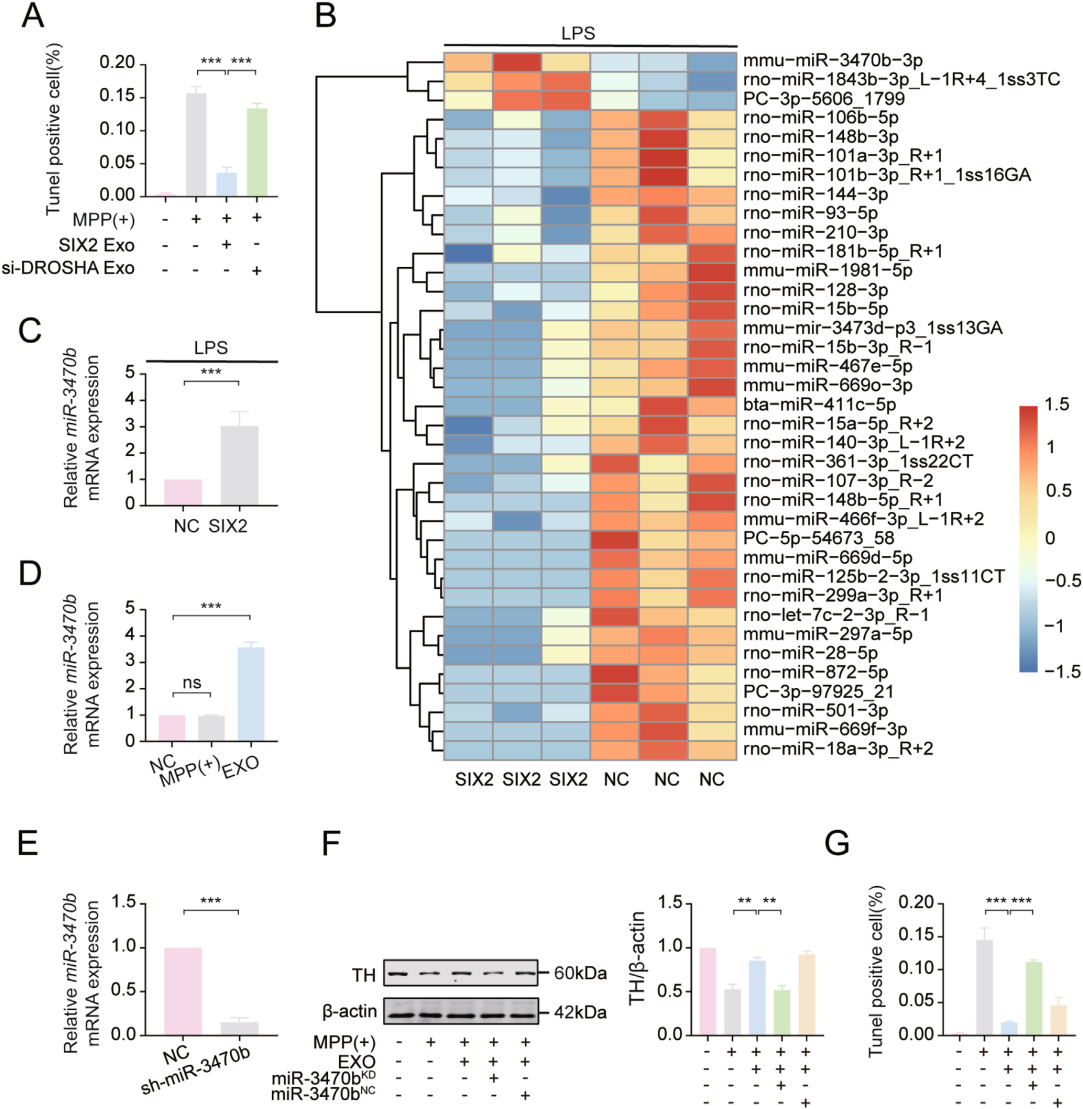
Exosomal miR‐3470b from LPS‐treated BV2_SIX2_ cells protects MES23.5 DA cells from MPP(+) damage. (A) TUNEL analysis of apoptosis in MPP(+)‐treated MES23.5 cells after treatment with exosomes from Drosha‐knockdown BV2_SIX2_ cells (LPS‐treated), Scale bar = 100 μm. (B) Heatmap of differentially expressed miRNAs from miRNA sequencing in LPS‐treated BV2_SIX2_ cells. (C) RT‐qPCR analysis of miR‐3470b levels in exosomes from LPS‐treated BV2_SIX2_ cells. (D) RT‐qPCR analysis of miR‐3470b expression in MPP(+)‐treated MES23.5 cells and MES23.5 cells treated with BV2_SIX2_‐derived exosomes. (E) RT‐qPCR validation of miR‐3470b knockdown in BV2 cells. (F, G) Exosomes from miR‐3470b‐knockdown BV2_SIX2_ cells (LPS‐treated) were applied to MPP(+)‐treated MES23.5 cells. (F) WB analysis of TH expression. (G) TUNEL analysis of apoptosis, Scale bar = 100 μm. (*p < 0.05, **p < 0.01, ***p < 0.001, *n* = 3).

Consistent with these findings, TUNEL assays revealed that the protective effect of SIX2 overexpression against MPP(+)‐induced damage in MES23.5 cells was abolished by DROSHA knockdown (Figure 6A). These results strongly suggest that exosomes from LPS‐treated BV2_SIX2_ cells exert protective effects on MES23.5 cells via miRNA transfer. To identify specific miRNAs mediating this protective effect, miRNA sequencing was performed on exosomes from LPS‐treated BV2_SIX2_ cells (Figure 6B). Among the differentially expressed miRNAs, miR‐3470b was significantly upregulated and validated by RT‐qPCR (Figure 6C). Application of these exosomes to MPP(+)‐treated MES23.5 cells significantly increased miR‐3470b expression compared to controls, while MPP(+) treatment alone did not alter miR‐3470b levels (Figure 6D). This indicates that the increased miR‐3470b in MES23.5 cells resulted from exosomal transfer rather than endogenous upregulation.

To further validate the role of exosomal miR‐3470b, sh‐miR‐3470b virus was used to knockdown miR‐3470b in BV2_SIX2_ cells (Figure 6E). Exosomes from miR‐3470b‐knockdown BV2_SIX2_ cells were collected after LPS treatment and applied to MPP(+)‐treated MES23.5 cells. These exosomes significantly reduced TH expression (Figure 6F) and increased apoptosis (Figure 6G) in MES23.5 cells. Together, these results demonstrate that exosomal transfer of miR‐3470b from LPS‐treated BV2_SIX2_ cells protects MES23.5 cells from MPP(+)‐induced damage.

### 2.7 Exosomal miR‐3470b Protects MES23.5 Cells From MPP(+)‐Induced Damage by Targeting GREM1

**FIGURE 7:**
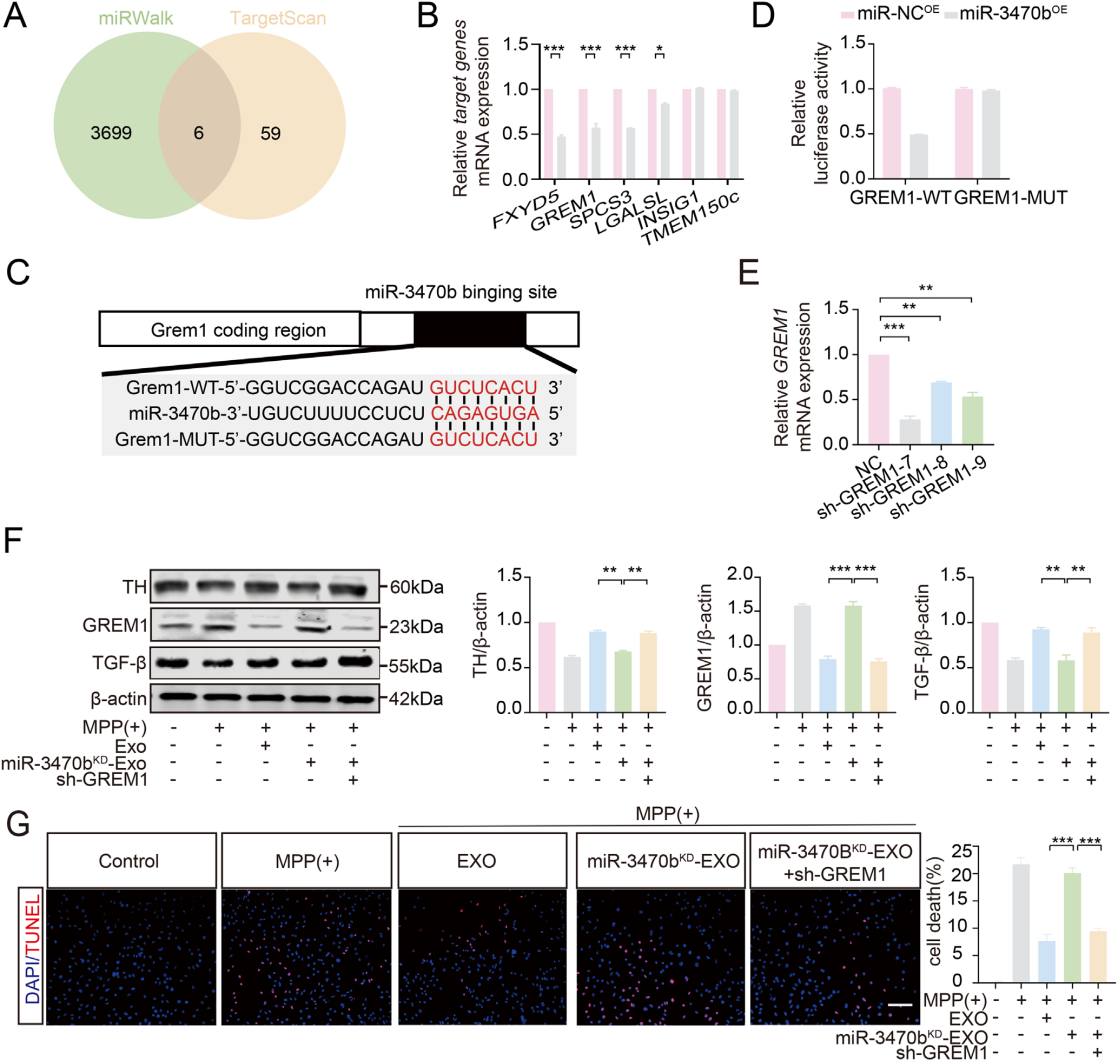
Role of miR‐3470b in regulating GREM1 and its impact on MES23.5 DA cells. (A) Venn diagram of miR‐3470b target genes predicted by miRWalk and TargetScan. (B) RT‐qPCR analysis of *FXYD5*, *GREM1*, and *SPCS3* levels in MPP(+)‐treated MES23.5 cells treated with exosomes from LPS‐treated BV2_SIX2_ cells. (C) Predicted binding site of miR‐3470b in the *GREM1* promoter region (TargetScan). (D) Dual‐luciferase assay evaluating miR‐3470b regulation of GREM1 in MES23.5 cells. (E) RT‐qPCR validation of *GREM1* knockdown efficiency in MES23.5 cells infected with sh‐GREM1 lentivirus (F, G) Exosomes from miR‐3470b‐knockdown BV2_SIX2_ cells (LPS‐treated) were applied to sh‐GREM1‐infected MES23.5 cells treated with MPP(+). (F) WB analysis of TH expression. (G) TUNEL analysis of apoptosis. Scale bar = 100 μm. (*p < 0.05, **p < 0.01, ***p < 0.001, *n* = 3).

Using miRWalk and TargetScan software, we identified the downstream target genes of miR‐3470b in MES23.5 cells. Venn analysis revealed the top 6 potential targets (Figure 7A). RT‐qPCR confirmed that exosomes from LPS‐treated BV2_SIX2_ cells significantly reduced the expression of *FXYD5*, *GREM*1, and *SPCS3* in MES23.5 cells (Figure 7B). Given the significant correlation between GREM1 and cell survival, we selected GREM1 for further investigation. To determine if miR‐3470b targets GREM1 in MES23.5 cells, we used TargetScan software to predict a binding site (CAGAGTGA) within the GREM1 3′‐UTR. Plasmids containing either the wild‐type or mutated (ACTCTGTC) binding site were constructed and co‐transfected with helper plasmids into MES23.5 cells. Mutation of the miR‐3470b binding site (CAGAGTGA to ACTCTGTC) in the GREM1 3′‐UTR significantly reduced luciferase activity, confirming GREM1 as a direct target of miR‐3470b in MES23.5 cells (Figure 7C,D).

To further validate these findings, sh‐GREM1 lentivirus was used to knock down GREM1 in MES23.5 cells, and the most effective lentivirus was selected (Figure 7E). miR‐3470b was then knocked down in LPS‐treated BV2_SIX2_ cells, and exosomes from these cells were applied to MES23.5 cells infected with sh‐GREM1 lentivirus and treated with MPP(+). Compared to exosomes from miR‐3470b‐knockdown BV2 cells, exosomes from cells with simultaneous miR‐3470b knockdown and GREM1 knockdown significantly increased TH expression and downstream TGF‐β signaling in MES23.5 cells (Figure 7F). TUNEL assays also showed reduced apoptosis in the simultaneous knockdown group (Figure 7G). These results demonstrate that exosomal miR‐3470b from LPS‐treated BV2_SIX2_ cells protects MES23.5 cells from MPP(+)‐induced damage by inhibiting GREM1 and activating TGF‐β signaling.

### 2.8 Exosomal miR‐3470b Protects Midbrain Dopaminergic Neurons in a PD Model via GREM1

**FIGURE 8:**
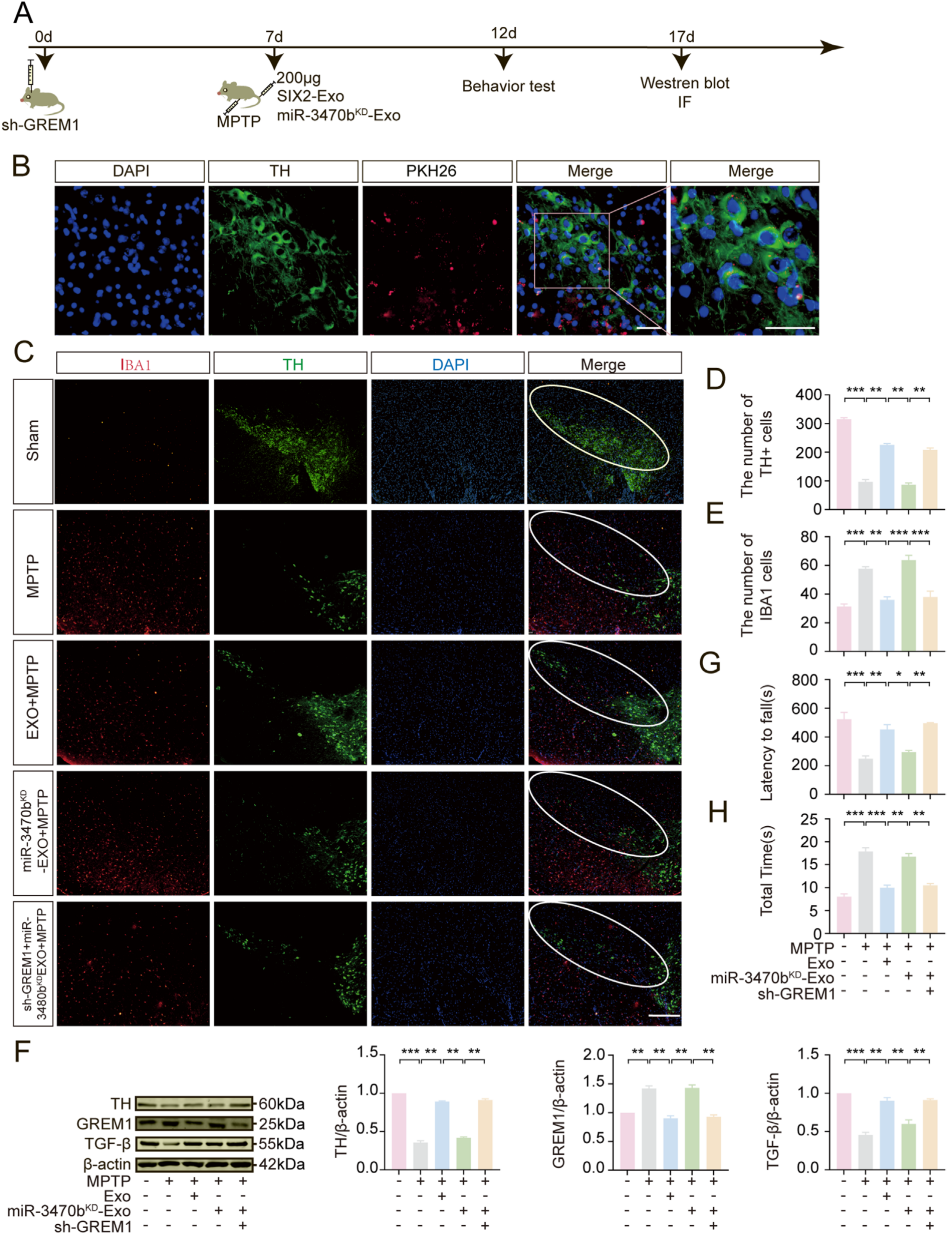
Exosomal miR‐3470b protects midbrain dopaminergic neurons in a PD model via GREM1. (A) Experimental flowchart: Sh‐GREM1 lentivirus was injected into the SNpc of mice, followed by intravenous administration of miR‐3470b^KD^‐Exo and intraperitoneal MPTP (30 mg/kg/day) injection to induce an acute PD model. (B) Immunofluorescence staining showing PKH26‐labeled exosomes (red) internalized by TH+ dopaminergic neurons (green), Scale bar = 50 μm. (C–E) Immunofluorescence analysis of TH+ dopaminergic neurons (green) and IBA1+ microglia (red) in the SNpc of PD mice, Scale bar = 200 μm. (F) WB analysis of TH, GREM1, TGF‐β expression in the midbrain of PD mice. (G, H) Motor function assessed by Rota‐Rod test (G) and Pole test (H). (*p < 0.05, **p < 0.01, ***p < 0.001, *n* ≥ 8).

To validate that exosomal miR‐3470b from LPS‐treated BV2_SIX2_ cells protects dopaminergic neurons by downregulating GREM1 in vivo, sh‐GREM1 lentivirus was injected into the SNpc of mice. One week later, two types of exosomes were injected intravenously: exosomes from LPS‐treated BV2_SIX2_ cells (Exo) and exosomes from miR‐3470b‐knockdown BV2_SIX2_ cells (miR‐3470b^KD^‐Exo). Concurrently, MPTP was administered intraperitoneally to induce an acute PD model (Figure 8A). To assess exosome delivery efficiency, immunofluorescence staining confirmed that intravenously administered exosomes successfully targeted and entered midbrain dopaminergic neurons (Figure 8B).

Further analysis of the SNpc showed a marked decrease in TH+ dopaminergic neurons in the MPTP group compared to controls. Exosome administration (Exo group) significantly increased TH+ neuron counts relative to the MPTP group. However, miR‐3470b knockdown (miR‐3470b^KD^‐Exo) significantly reduced TH+ neuron counts compared to the Exo group. Notably, simultaneous knockdown of miR‐3470b and GREM1 restored TH+ neuron counts to levels higher than those observed with miR‐3470b knockdown alone (Figure 8C,D). Conversely, IBA1+ microglia counts in the SNpc showed an inverse pattern (Figure 8C–E), suggesting a link between microglial activation and dopaminergic neuron health.

WB analysis revealed that exosome injection reversed MPTP‐induced downregulation of TH and TGF‐β while promoting GREM1 overexpression. This protective effect was abolished by miR‐3470b knockdown but restored by simultaneous GREM1 knockdown (Figure 8F). Behavioral tests, including the Rota‐Rod and climbing tests, further confirmed that exosomal miR‐3470b mitigates MPTP‐induced damage, an effect dependent on GREM1 suppression (Figure 8G,H). Together, these findings demonstrate that exosomal miR‐3470b from LPS‐treated BV2_SIX2_ cells infiltrates dopaminergic neurons, suppresses GREM1 expression, and protects against MPTP‐induced damage.

## 3 Discussion

Our study elucidates the pivotal role of SIX2 in regulating microglial polarization, a critical process in neuroinflammation associated with PD. Specifically, we demonstrate that SIX2 overexpression in microglial cells promotes their polarization toward an anti‐inflammatory M2 phenotype, essential for dopaminergic cell protection. Through a series of in vitro and in vivo experiments, we identified a novel molecular mechanism underlying this protective effect. First, we found that SIX2 directly upregulates DDIT4 expression by binding to its promoter region, leading to the suppression of mTOR signaling and subsequent activation of autophagy. This autophagy induction facilitates the transition of microglial cells from a pro‐inflammatory M1 phenotype to the neuroprotective M2 phenotype. Additionally, we discovered that M2‐polarized microglial cells secrete exosomes containing miR‐3470b, which are taken up by dopaminergic cells. Within these cells, miR‐3470b targets the GREM1/TGF‐β signaling pathway, thereby exerting significant neuroprotective effects. These findings not only highlight the dual role of SIX2 in modulating microglial polarization and neuronal survival but also provide new insights into the therapeutic potential of targeting the SIX2‐DDIT4‐miR‐3470b axis in PD.

Modulating the transition of microglial phenotypes from M1 to M2 is a promising therapeutic strategy for central nervous system repair [10, 30, 31]. In this study, we demonstrated that the transcription factor SIX2 plays a significant role in this process. We observed that endogenous SIX2 expression increased in microglial cells after LPS treatment, and elevated levels of SIX2 promoted microglial polarization toward the M2 phenotype, thereby inhibiting LPS‐induced neuroinflammation and protecting dopaminergic cells. To further validate the role of SIX2, we utilized an AAV‐DIO‐SIX2 to specifically overexpress SIX2 in microglia of Cx3cr1‐CreERT2 mice. This overexpression also facilitated M2 polarization in the SNpc within an LPS‐induced mouse model. These findings indicate that SIX2 acts as an endogenous protective factor, countering damage from external stressors through its stress‐induced upregulation. Consistent with our earlier findings that SIX2 in microglia suppresses LPS‐induced neuroinflammation. Importantly, in that same study, we provided direct histological evidence that microglia‐specific SIX2 overexpression protects nigral dopaminergic neurons from LPS‐induced loss, as assessed by tyrosine hydroxylase (TH) staining [16]. Another study reported that SIX2 re‐expression in LPS‐treated macrophages exhibited potent anti‐inflammatory effects by inhibiting the non‐canonical NF‐κB signaling pathway [15]. Together, these findings reinforce the notion that SIX2 is a critical factor in neuroprotection and inflammation modulation.

To elucidate the mechanism by which SIX2 promotes microglial polarization to the M2 phenotype, we conducted RNA‐Seq analysis combined with ChIP‐Atlas online analysis. Our findings revealed that SIX2 positively regulates *DDIT4* mRNA levels. DDIT4, a stress‐induced protein, regulates cellular metabolism and inflammation [32, 33, 34]. Importantly, DDIT4 has been shown to inhibit inflammatory responses by modulating oxidative stress and promoting metabolic remodeling [35]. In our study, we demonstrated that SIX2 upregulates DDIT4 expression by binding to the −901 bp to −886 bp region of the Ddit4 promoter. This upregulation of DDIT4 is a key step in facilitating microglial polarization to the M2 phenotype, ultimately protecting dopaminergic cells from inflammatory damage.

DDIT4 is a well‐established endogenous inhibitor of the mTOR [27, 36], a crucial regulator of cellular autophagy [28, 37]. Autophagy plays a critical role in suppressing neuroinflammatory responses [38, 39, 40], and its impairment in microglia exacerbates neuroinflammation through NLRP3 inflammasome activation, leading to motor and cognitive deficits in PD models [41]. Agents such as LPS and α‐synuclein inhibit autophagy, driving microglia toward a pro‐inflammatory M1 phenotype and worsening neurodegeneration [42, 43, 44]. Conversely, enhancing autophagy promotes M2 polarization and neuronal repair [45, 46]. Notably, DDIT4 has been shown to enhance macrophage metabolic reprogramming by promoting autophagy, offering protection against immune‐driven pathologies such as nonalcoholic fatty liver disease and colonic inflammation [35]. In our study, we confirmed that DDIT4 inhibits mTOR activity, leading to the activation of autophagy. This autophagy activation promotes the polarization of microglia toward the M2 phenotype, ultimately protecting co‐cultured dopaminergic cells. These findings suggest that SIX2 regulates microglial polarization through the DDIT4/mTOR/autophagy axis, providing a mechanistic basis for its neuroprotective effects.

Subsequently, we investigated the mechanism by which M2‐polarized microglia, induced by SIX2 overexpression, protect dopaminergic cells. Focusing on exosomes as key mediators of intercellular communication, we discovered that exosomes derived from SIX2‐overexpressing microglia play a pivotal role in this process. Specifically, miR‐3470b was identified as a significantly upregulated miRNA in these exosomes, and we demonstrated its ability to promote the survival of dopaminergic cells. Although the precise mechanisms remain to be fully elucidated, existing studies suggest its importance in modulating inflammatory and degenerative processes. For instance, macrophage‐derived miR‐3470b has been shown to inhibit osteolysis by suppressing TAB3/NF‐κB signaling [47], suggesting its role in neuroinflammation and neuronal survival. These findings underscore the critical role of exosomal miR‐3470b in mediating the neuroprotective effects of SIX2‐overexpressing microglia, offering new insights into therapeutic strategies for PD.

To explore the specific mechanism underlying the protective role of miR‐3470b, we identified GREM1 as its downstream target. GREM1, the antagonist of TGF‐β, has been implicated in various pathological processes, including neurodegeneration [48, 49, 50]. TGF‐β is critical in numerous physiological and pathological processes [51, 52], and activating the TGF‐β pathway can protect dopaminergic neurons of PD [53, 54, 55, 56, 57]. In our study, we demonstrated that GREM1 inhibits TGF‐β signaling in dopaminergic cells, exacerbating cell injury. Importantly, exosomal miR‐3470b mitigates this detrimental effect by targeting GREM1, thereby restoring TGF‐β activity and promoting neuroprotection. These findings highlight the critical role of the miR‐3470b/GREM1/TGF‐β axis in mediating the neuroprotective effects of SIX2‐overexpressing microglia.

In summary, this study, together with our previous work, establishes the transcription factor SIX2 as an endogenous protective factor in central nervous system microglia that operates through a dual mechanism. While our prior research showed that SIX2 suppresses neuroinflammation by upregulating FXYD2 to inhibit the NF‐κB pathway [16], the present work reveals that SIX2 concurrently activates a reparative program. Specifically, SIX2 upregulates the DDIT4/mTOR signaling axis, which activates autophagy and facilitates the polarization of microglia from a pro‐inflammatory M1 phenotype to an anti‐inflammatory M2 phenotype. These M2‐polarized microglia then release exosomes containing miR‐3470b, which specifically target the GREM1/TGF‐β signaling pathway in dopaminergic neurons, protecting them in PD model mice. This study uncovers a new mechanism by which SIX2 regulates microglial polarization to M2, offering new targets for PD immunotherapy. It also introduces exosome‐based miR‐3470b delivery, laying a foundation for exosome‐mediated miRNA therapy.

### 3.1 Limitations

Despite the promising findings, this study has several limitations that should be acknowledged. First, the primary mechanistic insights were derived from in vitro cell line models, which may not fully recapitulate the complexity of the in vivo brain microenvironment. Second, the study focused on a specific set of pathways (autophagy and exosome release), and the potential involvement of other parallel mechanisms cannot be ruled out. Third, the mechanistic validation in this study has certain constraints. The role of SIX2 was examined primarily under inflammatory challenge, leaving the effect of SIX2 overexpression alone on basal autophagy flux and mTOR signaling to be defined. Furthermore, the pharmacological blockade with 3‐MA, while supporting the necessity of autophagy, would be strengthened in future work by the inclusion of a full set of controls (e.g., inhibitor‐alone groups) to formally exclude potential off‐target effects. Future studies employing cell‐type‐specific knockout models and chronic disease paradigms will be crucial to validate and extend our conclusions.

## 4 Materials and Methods

### 4.1 Experimental Animals

Cx3cr1‐CreERT2 mice and C57 mice (22–25 g) were housed at 40%–70% relative humidity and 23°C ± 1°C on 12 h (12 h) light/dark cycle with a standard diet and water. Only adult male mice were used in this study to avoid potential confounding effects of the estrous cycle on neuroinflammatory responses. After acclimatization, mice were randomly assigned to experimental groups based on body weight to ensure baseline homogeneity. The Animal Care and Use Committee of Xuzhou Medical University approved the investigation (approval No. IACUC‐2022095069). All animal experiments are conducted in accordance with ARRIVE guidelines.

### 4.2 Tamoxifen and Adeno‐Associated Virus (AAV) Treatment Protocol in Cx3cr1‐CreERT2 Mice

Tamoxifen (20 mg/mL, MCE, New Jersey, USA) was dissolved in corn oil. Starting at 8 weeks of age, Cx3cr1‐CreERT2 mice received tamoxifen (0.2 mg/g body weight, i.p.) every other day for a total of five doses. One week later, the mice were injected with 300 nL of AAV‐NC or AAV‐SIX2, purchased from BrainVTA (Wuhan, China), into the right SNpc at the following coordinates: anteroposterior −3.3 mm, lateral 1.2 mm, dorsoventral −4.6 mm. Four weeks after the AAV injection, 1 μL of lipopolysaccharide (LPS, 5 μg/μL) or PBS was injected into the right SNpc of the mice. After surgery, the mice were placed in an initial incubator to recover before being returned to their cages.

### 4.3 Injection of Sh‐GREM1 Lentivirus Into the SNpc of Mice

The mice were anesthetized with pentobarbital (50 mg/kg) and then injected with 2 μL of sh‐GREM1 lentivirus into the SNpc (anteroposterior −3.3 mm, lateral ±1.2 mm, dorsoventral −4.6 mm), which was purchased from Jikai Gene Medical Technology (Shanghai, China). One week later, an intraperitoneal injection of MPTP was administered to establish a PD model.

### 4.4 Construction of MPTP Mouse Model and Tail Vein Injection of Exosomes

Mice received MPTP (30 mg/kg) [58] or an equal amount of 0.9% saline for 5 consecutive days. Neuronal death was assessed by immunofluorescence staining 5 days after MPTP injection. To perform the tail vein injection, hold and pull the tail with one hand. With the other hand, insert the needle (bevel facing up) into the lateral vein approximately 2–3 cm from the trunk of the mouse. The needle should be at a 30–45 degree angle to the plane of the tail. As the needle enters the vein, adjust it to a position almost parallel to the tail and push it forward to insert most of the needle into the vein. After injecting 200 μg [59] of exosomes, slowly withdraw the needle and apply pressure to the injection site to stop any bleeding. The presence of exosomes in the brain was detected by immunofluorescence staining 3 days after injection.

### 4.5 Cell Culture

Mouse BV2 microglia cells (CL‐0493) and 293 T cells (CL‐0005) were obtained from pricella (Wuhan, China), and murine dopaminergic cell line MES23.5 dopaminergic cells (HTX2826) were obtained from otwobiotech (Shenzhen, China). Cells were cultured in Dulbecco's Modified Eagle's Medium (DMEM) High glucose or F12 (KeyGEN Bio TECH, Nanjing, China) in the 5% CO_2_, 37°C incubator, which contained fetal bovine serum (FBS, 10%) (Clark, Richmond, USA) and penicillin/streptomycin (PS, 1%) (Beyotime Biotechnology, Shanghai, China). LPS was purchased from Sigma‐Aldrich (L6529, St. Louis, USA). 3‐MA (5 mM) and Bafilomycin A1 (100 nM) [60] were purchased from MCE (HY‐19312/HY‐100558, New Jersey, USA).

The final working concentrations, treatment durations, and references for all key reagents used in cell‐based assays are detailed in Table S1.

### 4.6 Primary Microglia Isolation From Postnatal Rat Brains

Glail cells were generated from rat pups 1–3 days after birth. Pups' brains were extracted and placed in the ice‐cold DMEM/F12 (KeyGEN Bio TECH, Nanjing, China). The meninges of brain tissue were removed, and the brain tissue was chopped into little bits with scissors. The tissue was dissociated with 0.125% trypsin in a 5%, 37°C incubator for 12 min (12 min). The enzymatic reaction was then halted by fetal bovine serum (10%, Clark, Richmond, USA). The mixture was filtered through a cell strainer (70 μm). After centrifugation (1200 rpm, 5 min, room temperature), the cells were seeded in a T25 flask and nurtured in the incubator. After 5 days, when astrocytes adhered to the bottom of the flask at 100% confluency, microglia were isolated by shaking. Microglia were collected for further experiments.

### 4.7 Lentivirus Infection

SIX2 lentivirus for overexpression and knockdown was obtained from Yunzhou Biologics (Guangzhou, China). DDIT4 lentivirus for overexpression was obtained from Obio Technology (Shanghai, China). GREM1 and miR‐3470b lentiviruses for knockdown were obtained from Jikai Gene Medical Technology (Shanghai, China). BV2 microglial cells and MES23.5 dopaminergic cells were transfected with lentiviruses according to the instructions and cultured with puromycin (4 μg/mL, Meilunbio, Dalian, China) after 72 h.

### 4.8 Small Interfering RNA (siRNA) Transfection

SiRNA of DDIT4, Drosha, and GP‐transfect‐Mate were obtained from GenePharma (Suzhou, China). BV2 microglial cells were cultured in 6‐plate and transfected according to the guidance. The sequences of DDIT4 and Drosha siRNA sense and anti‐sense are as follows:

**Table:** 

Gene name	Primer sequence (5′‐3′)
DDIT4‐siRNA‐581	Sense GCA AGG CAA GAG CUG CCA UTT Anti‐sense AUG GCA GCU CUU GCC UUG CTT
DDIT4‐siRNA‐676	Sense GCC UCU GGC CCA AGA UCC ATT Anti‐sence UGG AUC UUG GGC CAG AGG CTT
DDIT4‐siRNA‐763	Sense GCU UCA GAG UCA UCA AGA ATT Anti‐sence UUC UUG AUG ACU CUG AAG CTT
Scrambled siRNA	Sense UUC UCC GAA CGU GUC ACG UTT Anti‐sence ACG UGA CAC GUU CGG AGA ATT
DROSHA‐siRNA‐3223	Sense CCACCUGUACUACCUGUUUTT Anti‐sence AAACAGGUAGUACAGGUGGTT
DROSHAa‐siRNA‐4300	Sense GCGGUUCAUUGAGCGGAAATT Anti‐sence UUUCCGCUCAAUGAACCGCTT
DROSHA‐siRNA‐2686	Sense CCUAGCAAACAGUCCCAAATT Anti‐sence UUUGGGACUGUUUGCUAGGTT

### 4.9 Exosome Isolation and Identification From BV2 Cells

Exosomes were prepared from BV2 cell culture. The medium was collected and centrifuged at 300 *g* for 10 min, 2000 *g* for 10 min, and 10,000 *g* for 30 min at 4°C. Following centrifugation, the supernatant was passed through a 0.22 μm sterile filter (NEST, USA). The filtered supernatant was then transferred to a centrifuge tube and underwent a centrifugation step at 170,000 *g* for 120 min at 4°C in an Optima XPN‐100 Ultracentrifuge (Beckman Coulter) to purify the exosomes. Exosomes were resuspended in 20 μL PBS and stored at −80°C for subsequent experiments.

Nanoparticle tracking analysis (Zetaview, Germany) was used to analyze the distribution of vesicle diameters from the exosomes. A transmission electron microscope (TEM; Tecnai G2 Spirit Twin, USA) was used to observe the morphology of the acquired exosomes. Western blot analysis was performed to determine specific exosome surface markers, such as CD9, CD63, TSG101, and GRASP65.

### 4.10 Exosome Uptake by MES23.5 Cells

Fluorescent exosome labeling was performed according to the manufacturer's instructions. A 4 mg/mL PKH26 solution (Sigma‐Aldrich, St. Louis, MO) was added to PBS containing exosomes and incubated. Excess dye from the labeled exosomes was removed by ultracentrifugation at 170,000 *g* for 1 h at 4°C. The PKH26‐labeled exosomes were co‐cultured with MES23.5 cells for 24 h, after which the cells were washed with PBS and fixed in 4% paraformaldehyde. The uptake of Dil‐labeled exosomes by MES23.5 cells was observed using an Olympus fluorescence microscope.

### 4.11 Quantitative Real‐Time PCR (RT‐qPCR)

The RNA was extracted and subsequently reverse‐transcribed into cDNA, using a TIANGEN kit from Beijing, China. The qPCR assays were carried out using the Roche 480 real‐time PCR machine, with the universal SYBR Green Fast qPCR Mix provided by Vazyme (Nanjing, China). The relative mRNA expression was determined based on the 2^−ΔΔCt^ calculation method. The associated primers are as follows:

**Table:** 

Gene name	Primer sequence (5′‐3′)
TNF‐α	F: 5′ CCTGTAGCCCACGTCGTAG 3′ R: 5′ GGGAGTAGACAAGGTACAACCC 3′
IL‐6	F: 5′ CTGCAAGAGACTTCCATCCAG 3′ R: 5′ AGTGGTATAGACAGGTCTGTTGG 3′
iNOS	F: 5′ GGAGTGACGGCAAACATGACT 3′ R: 5′ TCGATGCACAACTGGGTGAAC 3′
ARG‐1	F: 5′ CTCCAAGCCAAAGTCCTTAGAG 3′ R: 5′ AGGAGCTGTCATTAGGGACATC 3′
IL‐10	F: 5′ GCTCTTACTGACTGGCATGAG 3′ R: 5′ CGCAGCTCTAGGAGCATGTG 3′
CD206	F: 5′ CTTCGGGCCTTTGGAATAAT 3′ R: 5′ TAGAAGAGCCCTTGGGTTGA 3′
DDIT4	F: 5′ GTGCTGCGTCTGGACTCTC 3′ R: 5′ CCGGTACTTAGCGTCAGGG 3′
GAPDH	F: 5′ AGGTCGGTGTGAACGGATTTG3′ R: 5′ TGTAGACCATGTAGTTGAGGTCA 3′
miR‐3470b	F: 5′ CCGATCGTTCCCCTCCATACAA 3′ R: 5′ ATTGCCTCTGCCTCCCAA 3′
GREM1	F: 5′ GCACAATGACTCCGAGCAGAC 3′ R: 5′ GACTCAAGCACCTCCTCTCCAG 3′
FXYD5	F: 5′ AGCCACAGGAAGCCAGACAG 3′ R: 5′ ACCTTTCTCGCTGCTTGTAAGTG 3′
SPCS3	F: 5′ TTTCATCACCACCGCCTTCAAAG 3′ R: 5′ TCCAAGGTCACTTCTTTCTCTAGGG 3′
LGALSA	F: 5′ TGAAGATCCTCCTGCCGATGTG 3′ R: 5′ AACGGAAAGTAAGGGATTGCTGAC 3′
INSIG1	F: 5′ GGCGTGGTCCTGGCTCTG 3′ R: 5′ CGGAGGAGAAGATGGTGGCTATC 3′
TMEM150C	F: 5′ CCTTTGGGTTTGGCACACTGAC 3′ R: 5′ GATGAAATACAGGACCACGCAGAG 3′
DROSHA	F: 5′ AGGATGGAATTTCTGGGCGACTC 3′ R: 5′ TTGGCTTGCGTTCTGTTGTTCAC 3′

### 4.12 Western Blot (WB)

The proteins from BV2 microglial cells, MES23.5 dopaminergic cells, and mouse midbrains were extracted using RIPA buffer (Beyotime Biotechnology, Shanghai, China), which contained protease and phosphatase inhibitor cocktails (Vicmed, Xuzhou, China). Protein separation was performed using the PAGE Gel Quick Preparation Kit (Yeasen, Shanghai, China), with 40 μg of protein for BV2 microglial cells and 60 μg for tissue samples. The proteins were then transferred to a nitrocellulose membrane (PALL/Bolida, Xuzhou, China), which was subsequently blocked with 5% nonfat milk (Solarbio, Beijing, China). The nitrocellulose membranes were treated with primary antibodies for 12 h at 4°C, followed by incubation with secondary antibodies (LI‐COR, Lincoln, USA) for 1 h at room temperature. The following antibodies were used: anti‐p62 (1:5000, 18,420‐1‐AP), anti‐CD63 (1:5000, 60,232‐1‐Ig), anti‐CD9 (1:5000, 60,232‐1‐Ig), anti‐TH (1:5000, 25,859‐1‐AP), anti‐TSG101 (1:500, 28,283‐1‐AP), anti‐GRASP65 (1:5000, 10,747‐2‐AP), and anti‐β‐actin (1:200,000, 66,009‐1‐Ig) from Proteintech (Wuhan, China); anti‐GREM1 (1:500, DF15419) from Affinity Biosciences (Melbourne, USA); anti‐TGF‐β (1:1000, orb89934) from biorbyt (Cambridge, UK); anti‐SIX2 (1:5000, ab111827), anti‐mTOR (1:3000, ab32028), and anti‐p‐mTOR (1:5000, ab109268) from Abcam (Cambridge, UK); anti‐DDIT4 (1:1000, A14135) from ABclonal (Wuhan, China), and anti‐iNOS (1:1000, 13,120), anti‐ARG‐1 (1:1000, 93,668), and anti‐LC3A/B (1:1000, 4108) from Cell Signaling Technology (Boston, USA). Data visualization was performed using the Odyssey imaging system (LI‐COR, Lincoln, USA) and processed with ImageJ software.

### 4.13 Enzyme‐Linked Immunosorbent Assay (ELISA)

IL‐10 and IL‐6 in the culture supernatant of BV2 microglial cells was measured with an ELISA kit (E‐EL‐M0046c, Elabscience, Wuhan, China). ELISA was carried out under the manufacturer's instructions. The optical density was measured using a microplate reader and evaluated using the standard curve.

### 4.14 Cell Viability Assay

After treating the BV2 microglial cells with LPS or PBS, they were centrifuged for 5 min at 1200 rpm. Following that, the supernatant was carefully aspirated and co‐incubated with MES23.5 dopaminergic cells for 24 h. Then, 10 μL CCK‐8 (Vicmed, Xuzhou, China) was added and cultured for 2 h. The data were obtained at 450 nm using a microplate reader (Synergy 2). Cell viability was normalized using the NC group.

### 4.15 TUNEL Analysis

TUNEL (Vazyme, Nanjing, China) was used to detect apoptosis in MES23.5 cells. The supernatant separated from BV2 microglial cells was co‐cultured with MES23.5 cells for 24 h. Following this, the cells were fixed at room temperature for 5 min before adding fluorescein‐labeled dUTP and incubating for 1 h at 37°C under the catalysis of terminal deoxynucleotide transferase. Fluorescence microscopy (Olympus) was then used to examine the labeled apoptotic cells.

### 4.16 Chromatin Immunoprecipitation (ChIP)

ChIP Assay Kit was obtained from Beyotime Biotechnology (Shanghai, China). The experiment was carried out according to the manufacturer's instructions. As previously described, BV2 microglial cells were treated with LPS (100 ng/mL) for 24 h. After treatment, cells were fixed for 10 min in 1% formaldehyde, followed by quenching with glycine and rinsing with cold PBS. Subsequently, cell nuclei were released and sonicated to generate 200–700 base pair (bp) DNA fragments. Aliquots of chromatin were incubated overnight with anti‐SIX2 antibody (4 μg, 11,562‐1‐AP, Proteintech, Wuhan, China) or rabbit IgG control antibody (4 μg, 30,000‐0‐AP, Proteintech, Wuhan, China). The chromatin‐antibody complexes were pulled down using prewashed magnetic beads (HY‐K0204, MCE, New Jersey, USA), then washed and eluted to recover DNA fragments associated with SIX2. Input genomic DNA was obtained through similar purification procedures. The binding amount was calculated using the enrichment factor. The primer sequences for qPCR are as follows:

**Table:** 

Gene name	Primer sequence (5′‐3′)
Sequence 1 (−901 bp to −886 bp)	F: 5′ GCC GTG AAA CTC CGT CCA AA 3′
	R: 5′ CAT CAC ATC GCT ACA GGG CA 3′
Sequence 2 (−618 bp to −603 bp)	F: 5′ TTT CCT GTT GCC TCG CCT G 3′
	R: 5′ CCT AGA ACC CCT CCC ATG CT 3′

### 4.17 Dual‐Luciferase Reporter Assay

The Dual‐LumiTM Luciferase Reporter Gene Assay kit was obtained from Beyotime Biotechnology (Shanghai, China). The promoter region of the mouse *DDIT4* and *GREM1* gene was subcloned into pGL3 Basic luciferase reporter plasmid (Sangon Biotech, Shanghai, China). Plasmids were transfected into 293 T cells. After 48 h, luciferase assays were carried out following the manufacturer's instructions. The relative light unit was measured by a microplate reader (Synergy 2).

### 4.18 Rota‐Rod Test

The degree of motor coordination in mice was assessed using a standard Rota‐Rod treadmill (Zhenghua Biologic, Anhui, China). Mice were acclimatized by being placed on the rotating bar for 20 min before the actual experiment. The initial speed of the Rota‐Rod was set to 5 rpm and gradually increased to 40 rpm over 10 min. Each mouse was placed on the rotating bar, and the duration it remained on it was recorded.

### 4.19 Pole Test

Following the Rota‐Rod test, a pole‐climbing test was conducted, where mice were trained for 3 days prior to acclimatization. The pole used was 50 cm in height. The experiment recorded the time taken for each mouse to climb continuously from the top to the bottom of the pole.

### 4.20 Immunofluorescence Staining

After behavioral testing, mice were anesthetized, and a needle was inserted into the left ventricle while the right atrial appendage was cut for perfusion. Continuous sections were made from the coronal surface of the mouse brain (bregma −3.87 mm to −2.69 mm) with a section thickness of 20 μm. Brain slices were washed, blocked for 1 h, and incubated with antibodies for 16 h at 4°C. The following antibodies were used: anti‐iNOS (1:1000, 13,120) from Cell Signaling Technology (Boston, USA), anti‐Iba‐1 (1:500, OB‐RB029, Asis Biofarm, Zhejiang, China), anti‐TNF‐α (1:100, sc‐24,723, Santa Cruz, Dallas, USA), anti‐CD206 (1:400, 60,143‐1‐Ig), anti‐TH (1:200, 25,859‐1‐AP), anti‐CD63 (1:200, 60,232‐1‐Ig), CoraLite Plus 488 (PF00001) from Proteintech (Wuhan, China). After washing, the slices were incubated for 1 h at room temperature with goat anti‐rabbit secondary antibody (1:400, ab150080, Abcam, Cambridge, UK) and goat anti‐mouse secondary antibody (1:400, ab150113, Abcam, Cambridge, UK). Images were captured using an Olympus fluorescence microscope.

### 4.21 Stereology

The number of tyrosine hydroxylase‐positive (TH+) neurons in unilateral brain sections of the mouse SNpc was quantified using ImageJ software (bregma −3.87 mm to −2.69 mm). The first brain section was randomly selected from the frontal aspect of the SNpc, with subsequent sections taken every five slices (20 μm each). Images were captured using an Olympus fluorescence microscope. TH+ neurons were counted only if they were completely or partially within an elliptical frame. The total number of TH+ neurons was obtained by summing the counts from each section of the SNpc.

### 4.22 Statistical Analysis

Data analysis was performed using GraphPad Prism 9 software. All experimental results are expressed as mean ± standard error of the mean (Mean ± SEM). For comparisons between two groups, independent samples *t*‐test was used; for multiple groups, one‐way analysis of variance (ANOVA) was conducted, followed by post hoc tests (e.g., Tukey's test) when appropriate to assess intergroup differences. A *p*‐value of < 0.05 was considered statistically significant. All experiments were repeated at least three times to ensure the reliability and reproducibility of the results.

## Author Contributions

Xia‐yin Cao and Jia‐shuo Kan designed and performed the major experiments, analyzed data, and interpreted the results. Yu‐xin Ye and Xin‐xing Huang provided technical support for cell culture, RNA‐seq, and miRNA sequencing experiments. Guo‐jing Sun contributed to the animal model experiments and behavioral tests. Jin Gao supervised the study, contributed to the conception and design, and wrote and revised the manuscript. All authors discussed and formulated the manuscript.

## Funding

This work was supported by the Natural Science Foundation of Jiangsu Province (grant number BK20221225), National Natural Science Foundation Project (grant number 81901299), Scientific Research Project of Affiliated Hospital of Xuzhou Medical University (2021ZA39), and Key Laboratory Open Research Project of Universities in Jiangsu Province at Xuzhou Medical University (XZSYSKF2024017).

## Ethics Statement

All animal experiments were conducted in accordance with the National Institutes of Health Guide for the Care and Use of Laboratory Animals and were approved by the Animal Care and Use Committee of Xuzhou Medical University (Approval ID: No. IACUC‐2022095069).

## Conflicts of Interest

The authors declare no conflicts of interest.

## Supporting information


**Figure S1:** Characterization of primary microglia and analysis of SIX2 expression. (A) Immunofluorescence analysis of primary microglia isolated from newborn SD rats using Iba‐1 antibody. (B) Fluorescence intensity analysis of SIX2 in primary microglia treated with LPS for 24 h. PMM:primary microglia model (**p < 0.01, *n* = 3).

**Figure S2:** Validation of SIX2 expression levels in BV2 microglial cells following knockdown or overexpression. (A) WB analysis of SIX2 expresison in BV2_shSIX2_ microglial cells. (B) WB analysis of SIX2 expresison in BV2_SIX2_ microglial cells (*p < 0.05 and **p < 0.01, *n* = 3).

**Figure S3:** SIX2 modulates the secretion of M1 and M2 cytokines in LPS‐activated BV2 microglia. (A) IL‐10 and (B) IL‐6 levels in the culture supernatant were measured by enzyme‐linked immunosorbent assay (ELISA) after SIX2 knockdown or overexpression followed by LPS (100 ng/mL) treatment for 24 h (*p < 0.05 and **p < 0.01, ***p < 0.001, *n* = 3).

**Figure S4:** Differential gene expression analysis and overlap with SIX2‐regulated targets in LPS‐treated microglia. (A)Volcano plot illustrating differentially expressed genes from RNA‐sequencing analysis of LPS‐treated BV2_shSIX2_ cells. (B) Venn diagram showing overlap between SIX2‐regulated target genes (ChIP‐atlas) and differentially expressed genes (RNA‐sequencing).

**Figure S5:** SIX2 promotes M2 polarization of LPS‐induced BV2 microglial cells by upregulating DDIT4. (A) WB analysis of DDIT4 expression in LPS‐treated BV2 cells overexpressing SIX2. (B) WB analysis of DDIT4 knockdown efficiency using three si‐DDIT4 constructs in BV2 cells. (C) RT‐qPCR validation of DDIT4 knockdown in BV2 cells. (D) WB analysis of SIX2 expression in primary microglia after SIX2 overexpression and DDIT4 silencing. (E) Immunofluorescence analysis of DDIT4 expression in primary microglia. (F) Immunofluorescence staining of F‐actin (green) to assess somatic size of LPS‐treated primary microglia, Scale bar = 10 μm. (G) Immunofluorescence staining of iNOS in BV2 cells overexpressing SIX2 and knocking down DDIT4 after LPS treatment, Scale bar = 50 μm. (H) Immunofluorescence staining of ARG‐1 in BV2 cells overexpressing SIX2 and knocking down DDIT4 after LPS treatment, Scale bar = 50 μm (* *p* < 0.05 and ** *p* < 0.01, *n* = 3).

**Figure S6:** Effects of DROSHA siRNA on TH expression in MPP(+)‐treated MES23.5 cells. (A) RT‐qPCR analysis of miRNA production in BV2 cells after DROSHA siRNA treatment. (B) WB analysis of TH expression in MPP(+)‐treated MES23.5 cells treated with exosomes from *DROSHA*‐knockdown BV2_SIX2_ cells (LPS‐treated) (**p < 0.01 and ***p < 0.001, *n* = 3).

**Table S1:** Summary of key reagents and treatment protocols.

## Data Availability

Data will be made available on request.
