# Supplementary figures and images for: SIX2‐Mediated Microglial M2 Polarization and Exosomal miR‐3470b Delivery Protect Dopaminergic Neurons in Parkinson's Disease
Source: CNS Neurosci Ther. 2026 Feb 17;32(2):e70756. doi: 10.1002/cns.70756 (PMC12910405; doi:10.1002/cns.70756)

Fig.S1

A

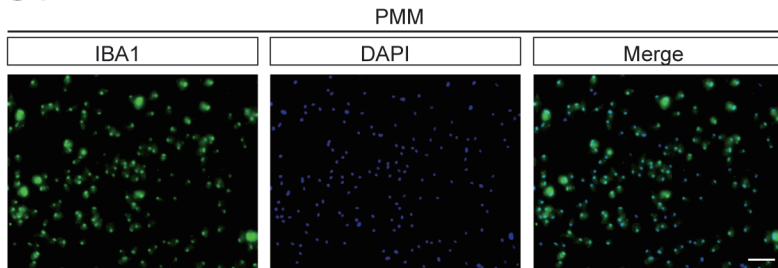

IBA1 positive cells  
other cells

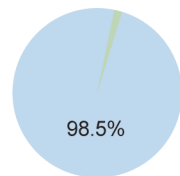

B

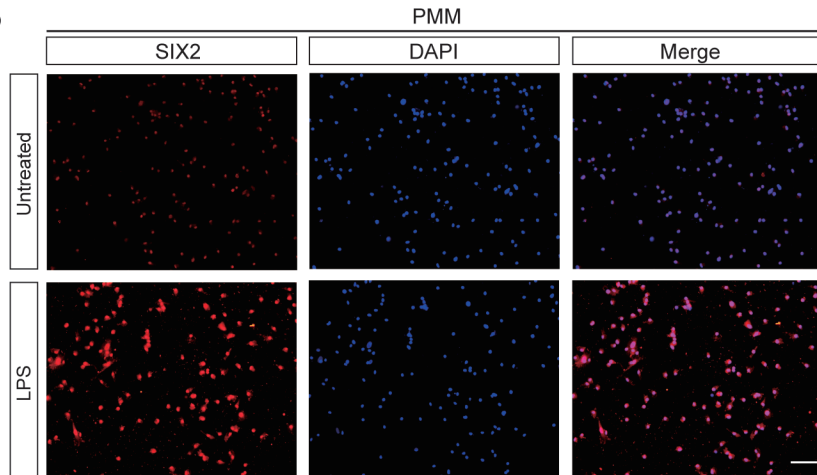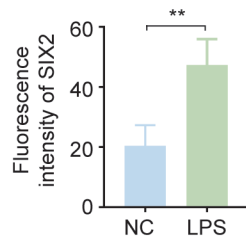

Supplement: Supplementary file 1 — Figure S1: Characterization of primary microglia and analysis of SIX2 expression. (A) Immunofluorescence analysis of primary microglia isolated from newborn SD rats using Iba‐1 antibody. (B) Fluorescence intensity analysis of SIX2 in primary microglia treated with LPS for 24 h. PMM:primary microglia model (**p < 0.01, n = 3). [file CNS-32-e70756-s004.pdf]

Fig.S2  
A

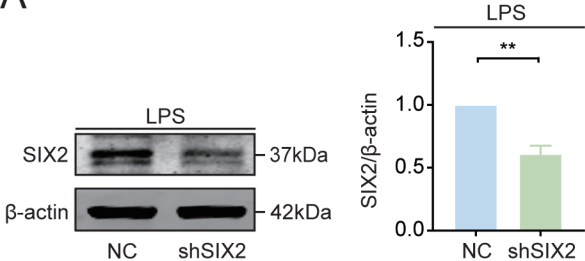

B

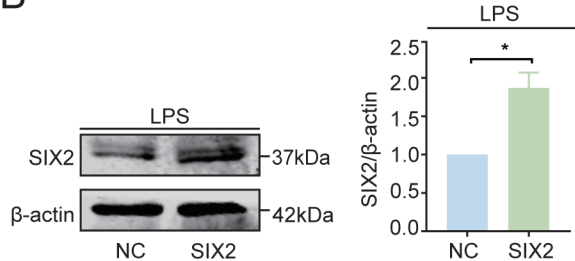

Supplement: Supplementary file 2 — Figure S2: Validation of SIX2 expression levels in BV2 microglial cells following knockdown or overexpression. (A) WB analysis of SIX2 expresison in BV2shSIX2 microglial cells. (B) WB analysis of SIX2 expresison in BV2SIX2 microglial cells (*p < 0.05 and **p < 0.01, n = 3). [file CNS-32-e70756-s003.pdf]

Fig.S3

A

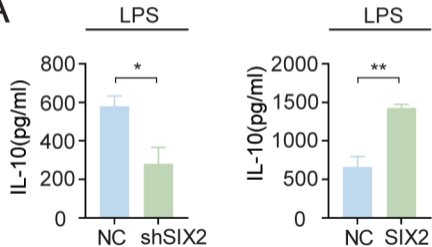

B

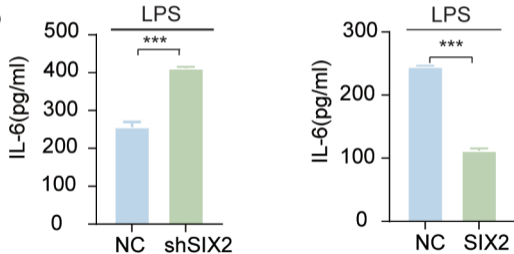

Supplement: Supplementary file 3 — Figure S3: SIX2 modulates the secretion of M1 and M2 cytokines in LPS‐activated BV2 microglia. (A) IL‐10 and (B) IL‐6 levels in the culture supernatant were measured by enzyme‐linked immunosorbent assay (ELISA) after SIX2 knockdown or overexpression followed by LPS (100 ng/mL) treatment for 24 h (*p < 0.05 and **p < 0.01, ***p < 0.001, n = 3). [file CNS-32-e70756-s001.pdf]

Fig.S4  
A

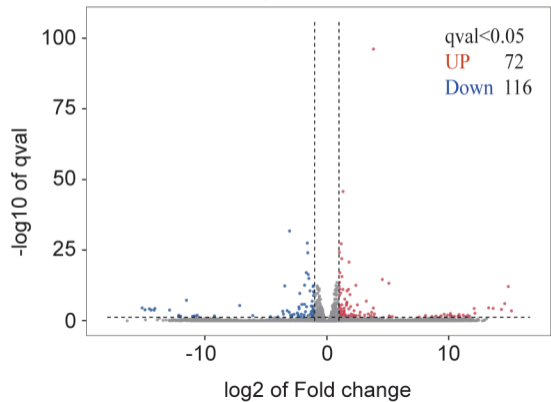

B

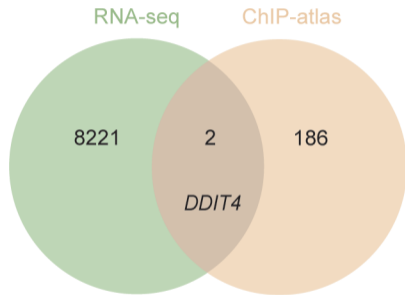

Supplement: Supplementary file 4 — Figure S4: Differential gene expression analysis and overlap with SIX2‐regulated targets in LPS‐treated microglia. (A)Volcano plot illustrating differentially expressed genes from RNA‐sequencing analysis of LPS‐treated BV2shSIX2 cells. (B) Venn diagram showing overlap between SIX2‐regulated target genes (ChIP‐atlas) and differentially expressed genes (RNA‐sequencing). [file CNS-32-e70756-s002.pdf]

Fig.S5

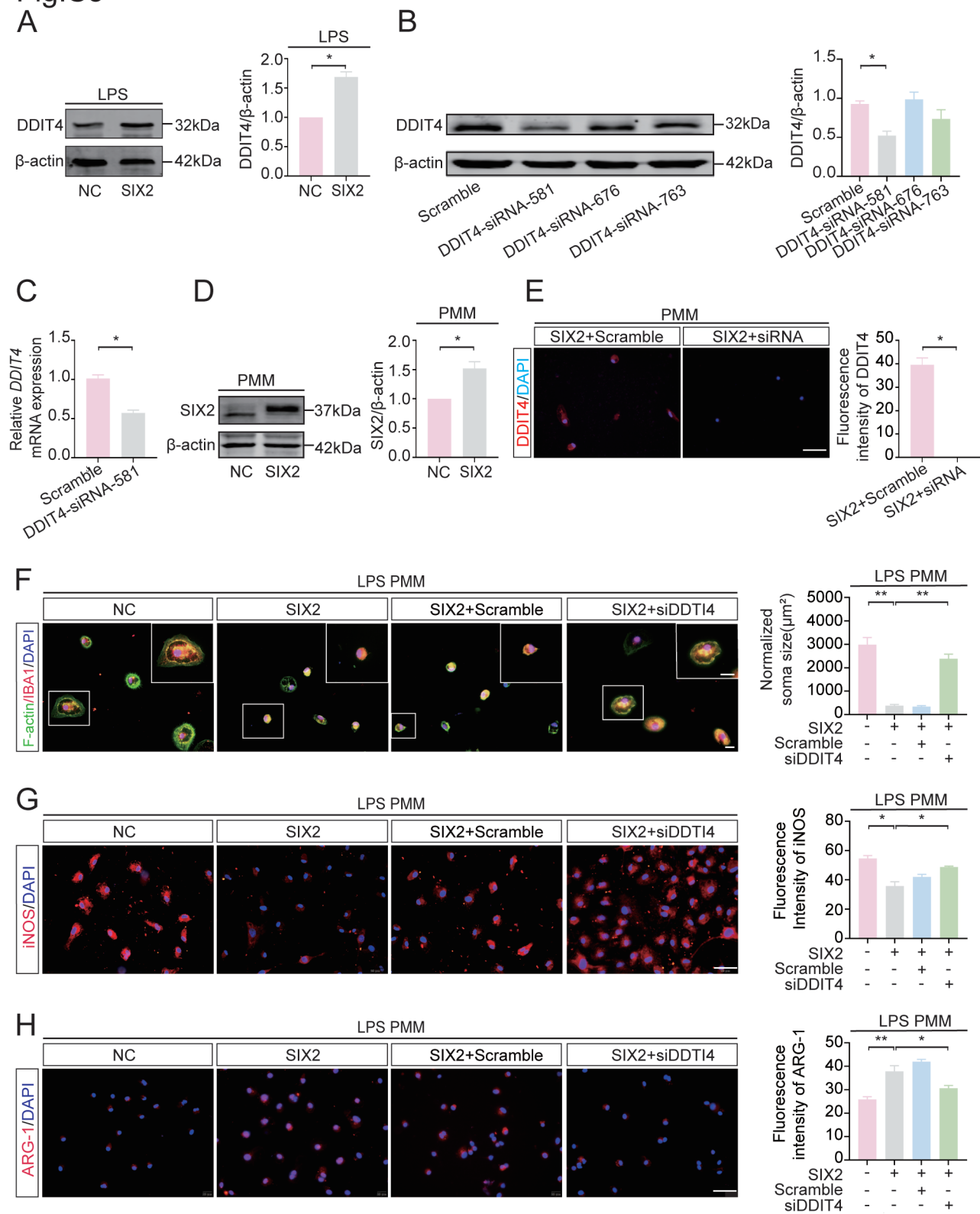

Supplement: Supplementary file 5 — Figure S5: SIX2 promotes M2 polarization of LPS‐induced BV2 microglial cells by upregulating DDIT4. (A) WB analysis of DDIT4 expression in LPS‐treated BV2 cells overexpressing SIX2. (B) WB analysis of DDIT4 knockdown efficiency using three si‐DDIT4 constructs in BV2 cells. (C) RT‐qPCR validation of DDIT4 knockdown in BV2 cells. (D) WB analysis of SIX2 expression in primary microglia after SIX2 overexpression and DDIT4 silencing. (E) Immunofluorescence analysis of DDIT4 expression in primary microglia. (F) Immunofluorescence staining of F‐actin (green) to assess somatic size of LPS‐treated primary microglia, Scale bar = 10 μm. (G) Immunofluorescence staining of iNOS in BV2 cells overexpressing SIX2 and knocking down DDIT4 after LPS treatment, Scale bar = 50 μm. (H) Immunofluorescence staining of ARG‐1 in BV2 cells overexpressing SIX2 and knocking down DDIT4 after LPS treatment, Scale bar = 50 μm (* p < 0.05 and ** p < 0.01, n = 3). [file CNS-32-e70756-s006.pdf]

Fig.S6

A

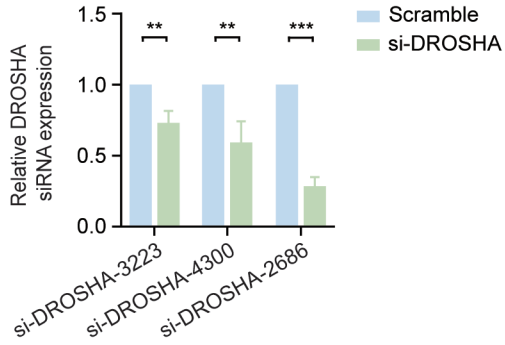

B

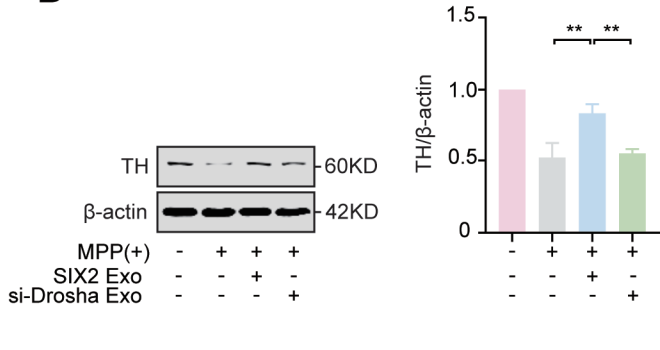

Supplement: Supplementary file 6 — Figure S6: Effects of DROSHA siRNA on TH expression in MPP(+)‐treated MES23.5 cells. (A) RT‐qPCR analysis of miRNA production in BV2 cells after DROSHA siRNA treatment. (B) WB analysis of TH expression in MPP(+)‐treated MES23.5 cells treated with exosomes from DROSHA‐knockdown BV2SIX2 cells (LPS‐treated) (**p < 0.01 and ***p < 0.001, n = 3). [file CNS-32-e70756-s007.pdf]
