# Supplementary material for: SIX2‐Mediated Microglial M2 Polarization and Exosomal miR‐3470b Delivery Protect Dopaminergic Neurons in Parkinson's Disease
Source: CNS Neurosci Ther. 2026 Feb 17;32(2):e70756. doi: 10.1002/cns.70756 (PMC12910405; doi:10.1002/cns.70756)
Supplement: Supplementary file 7 — Table S1: Summary of key reagents and treatment protocols. [file CNS-32-e70756-s005.docx]

**Supplementary Table S1. Summary of key reagents and treatment protocols.**

| **Purpose** | **Reagent** | **Model System** | **Treatment Details (Concentration/Dosage, Duration, Route)** | **Reference / Rationale** |
| --- | --- | --- | --- | --- |
| In Vitro Treatments | LPS | BV2 / Primary microglia | 100 ng/mL for 24 h | Reference # [46] |
|  | GW4869 | BV2 cells | 10 μM for 24 h | Reference # [30] |
|  | 3-MA | BV2 cells | 5 mM, pre-treatment for 2 h | Reference # [60] |
|  | BafA1 | BV2 cells | 100 nM, co-treatment for 2 h | Reference # [60] |
|  | MPP⁺ | MES23.5 cells | 300 μM for 24 h | Determined by pilot dose-response study |
|  | Exosomes | MES23.5 cells | 10 μg/mL for 24 h | Optimized for neuroprotection (Fig. 5I) |
| In Vivo Treatments | LPS | C57BL/6 mice | 0.25 mg/kg in 1 μL PBS, single i.c. injection | Reference # [16] |
|  | MPTP | C57BL/6 mice | 30 mg/kg/day for 5 consecutive days, i.p. | Reference # [58] |
|  | Exosomes | C57BL/6 mice | 200 μg in 100 μL PBS,twice a week, i.v. | Reference # [59] |
